# Approaching rational epitope vaccine design for hepatitis C virus with meta-server and multivalent scaffolding

**DOI:** 10.1038/srep12501

**Published:** 2015-08-04

**Authors:** Linling He, Yushao Cheng, Leopold Kong, Parisa Azadnia, Erick Giang, Justin Kim, Malcolm R. Wood, Ian A. Wilson, Mansun Law, Jiang Zhu

**Affiliations:** 1 Department of Immunology and Microbial Science, The Scripps Research Institute, La Jolla, California 92037, USA; 2 Department of Integrative Structural and Computational Biology, The Scripps Research Institute, La Jolla, California 92037, USA; 3 International AIDS Vaccine Initiative, Neutralizing Antibody Center and the Collaboration for AIDS Vaccine Discovery; 4 Center for HIV/AIDS Vaccine Immunology and Immunogen Discovery, The Scripps Research Institute, La Jolla, California 92037, USA; 5 Core Microscopy Facility, The Scripps Research Institute, La Jolla, California 92037, USA

## Abstract

Development of a prophylactic vaccine against hepatitis C virus (HCV) has been hampered by the extraordinary viral diversity and the poor host immune response. Scaffolding, by grafting an epitope onto a heterologous protein scaffold, offers a possible solution to epitope vaccine design. In this study, we designed and characterized epitope vaccine antigens for the antigenic sites of HCV envelope glycoproteins E1 (residues 314–324) and E2 (residues 412–423), for which neutralizing antibody-bound structures are available. We first combined six structural alignment algorithms in a “scaffolding meta-server” to search for diverse scaffolds that can structurally accommodate the HCV epitopes. For each antigenic site, ten scaffolds were selected for computational design, and the resulting epitope scaffolds were analyzed using structure-scoring functions and molecular dynamics simulation. We experimentally confirmed that three E1 and five E2 epitope scaffolds bound to their respective neutralizing antibodies, but with different kinetics. We then investigated a “multivalent scaffolding” approach by displaying 24 copies of an epitope scaffold on a self-assembling nanoparticle, which markedly increased the avidity of antibody binding. Our study thus demonstrates the utility of a multi-scale scaffolding strategy in epitope vaccine design and provides promising HCV immunogens for further assessment *in vivo*.

Hepatitis C is a global health burden with approximately 180 million chronic carriers of hepatitis C virus (HCV) worldwide^1^,^2^,^3^,^4^. Although small-molecule drugs are available for treatment^5^, a prophylactic vaccine will offer a cost-effective means to prevent HCV transmission and to eventually eradicate the virus^6^,^7^,^8^. However, due to the poor fidelity of the viral RNA polymerase^9^ and the high replication rate (10^12^ virions/day), HCV exhibits extraordinary genetic diversity with over 30% sequence difference between genotypes^10^, posing a significant challenge to the development of a broadly effective vaccine.

HCV entry is mediated by the interactions between viral envelope glycoproteins and cell surface receptors such as CD81, scavenger receptor class B type I (SR-BI), and other host entry factors^11^,^12^. While it is known that glycoproteins E1 and E2 form heterodimers on the surface of a virion, how they mediate viral membrane fusion and cell entry remain elusive^13^,^14^,^15^,^16^. Neutralizing antibodies (nAbs) induced during natural infection have been mapped onto both E1 and E2. Early studies in infected humans and chimpanzees showed that the highly variable region 1 (HVR1) located at the N-terminus of E2 (residues 384–410) is a major target for the antibody response^17^,^18^,^19^,^20^,^21^,^22^. Recently, several E2-directed broadly neutralizing antibodies (bnAbs) have been identified^23^,^24^,^25^,^26^,^27^. Structures of the E1 antigenic site (residues 314–324) in complex with antibody IGH526^28^,^29^ and the E2 antigenic site (residues 412–423) bound to murine and human bnAbs^26^,^30^,^31^,^32^,^33^,^34^ have revealed critical details of HCV epitope recognition by the humoral immune system. More recently, Kong *et al.* reported a 2.65 Å crystal structure of the E2 core domain (E2c) in complex with a human bnAb AR3C^35^, which showed a stark difference from the class II fusion protein model found in other *Flaviviridae* viruses^36^. This finding was confirmed by another E2c structure determined by Khan *et al.*^13^ It is foreseeable that this ever-accumulating structural information will enable an in-depth understanding of HCV biology as well as structure-based design of epitope vaccines.

Conventional approaches have failed to produce broadly effective vaccines for highly variable viral pathogens such as HCV and human immunodeficiency virus type-1 (HIV-1), which have evolved various mechanisms such as glycan shielding and immune decoys to evade host immunity^37^,^38^. Epitope grafting, or scaffolding, has been proposed as a possible solution to epitope vaccine design by transferring a conserved neutralizing epitope onto heterologous protein scaffolds^39^. For HIV-1, scaffold antigens have been designed for two linear epitopes in the membrane proximal external region (MPER) that are recognized by bnAbs 4E10^40^ and 2F5^41^, respectively. A large-scale *in-silico* design, coupled with high-throughput antigenic screening, was recently reported for three major HIV-1 epitopes, with up to 50% of the designs able to bind their respective bnAbs^42^. Scaffolding was also applied to the antigenic site II of the fusion (F) protein of respiratory syncytial virus (RSV)^43^, and neutralizing antibodies were induced by a scaffolded epitope in macaques^44^. Based on these studies, a general strategy consisting of epitope identification, scaffolding, particulate presentation of the designed antigen, animal immunization, and quantitative analysis of immune response has been proposed for epitope vaccine design and evaluation^45^.

In this study, we sought to develop robust scaffolding and particulate display methods for epitope vaccine design and with which to design epitope vaccine immunogens for the antigenic sites of HCV glycoproteins E1 (residues 314–324) and E2 (residues 412–423). We first developed a scaffolding meta-server by combining six structural alignment algorithms for scaffold search. We demonstrated increased coverage and diversity of the meta-server for an HIV-1 epitope and obtained a large number of scaffolds for the two HCV antigenic sites studied. We grafted the E1 and E2 antigenic sites onto twenty selected scaffolds, ten for each site, and analyzed the resulting antigens using residue-based quality scores and explicit-water molecular dynamics simulation. We characterized these epitope scaffolds by mammalian expression and antibody binding assays, with antigenicity confirmed for three E1 and five E2 designs. We then explored a multivalent scaffolding approach by displaying 24 copies of an epitope scaffold on a self-assembling ferritin nanoparticle. For both antigenic sites, the nanoparticles exhibited slow off-kinetics, suggesting a strong avidity effect due to multivalent antibody binding. We expect that the antigenic epitope scaffolds and nanoparticles will provide promising candidates for animal studies, while the new scaffolding methods will find many applications in epitope vaccine design.

## Results

### Development of a scaffolding meta-server

**Figure 1:**
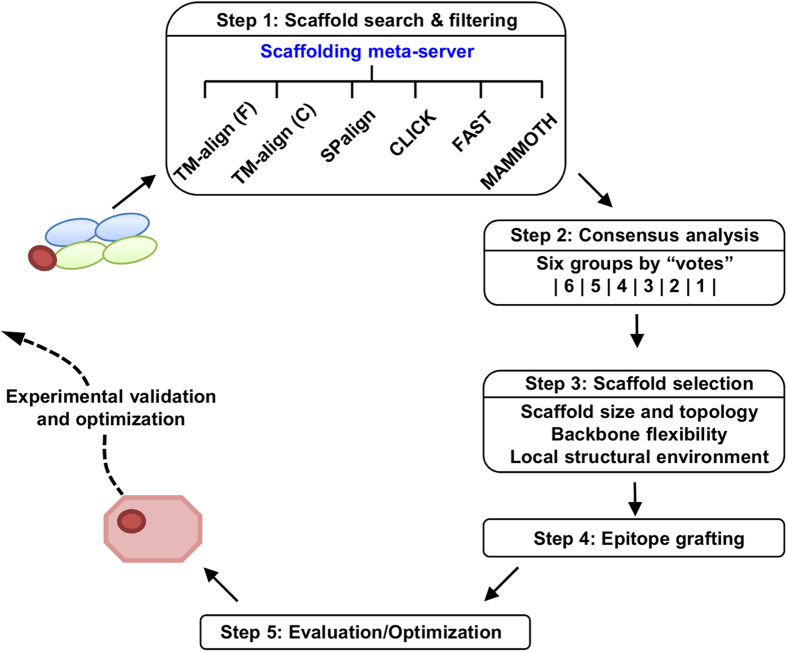
Schematic flow of an epitope scaffold design pipeline based on the scaffolding meta-server (step 1), a consensus-based analysis (step 2), scaffold selection (step 3), side chain-based epitope grafting (step 4), and evaluation and optimization of the designed antigens (step 5).

Scaffold search is the first step of epitope scaffold design, preceding epitope grafting and antigen optimization^45^. To date, most design studies have been conducted using a single structural alignment algorithm for scaffold search, which sampled only a subspace of protein structures in an algorithm-specific manner^40^,^41^,^42^,^43^,^44^,^46^,^47^. As a large number of structural alignment tools can be adapted for scaffolding, and meta-servers that combine output from multiple methods are widely accepted as a practical solution to difficult structure prediction problems^48^, we sought to develop a scaffolding meta-server to facilitate epitope vaccine design. We first examined the coverage of two previously reported scaffolding algorithms. Ofek *et al.* used MAMMOTH^49^ to search for scaffolds for the HIV-1 epitope recognized by 2F5^41^, whereas Zhou *et al.* utilized TM-align^50^ to identify scaffolds for three major HIV-1 bnAb epitopes^42^. In our benchmark test, MAMMOTH and TM-align identified 273 and 262 scaffolds for the 2F5 epitope, respectively, with a C_α_ root-mean-square deviation (RMSD) of 1.5 Å or lower in the epitope-matching region. Of these scaffolds, 79 are identical, suggesting that the two algorithms identified a set of different but overlapping scaffolds. We then included four more structural alignment algorithms—a C implementation of TM-align^50^, SPalign^51^, CLICK^52^, and FAST^53^, which identified 539, 331, 74, and 152 scaffolds, respectively (Fig. S1). Together, six scaffolding algorithms yielded 1,003 non-redundant scaffolds with a pairwise overlap of 22–202, indicating that a large protein structural space has been covered in the search. Our results thus illustrate the improved coverage by combining six scaffolding algorithms in a meta-server and a consensus-based approach for scaffold selection. An epitope scaffold design pipeline was devised based on the scaffolding meta-server (Fig. 1, see Methods) and applied to the two HCV antigenic sites.

### Epitope scaffold design for the E1 antigenic site 314–324

A 1.75 Å crystal structure was recently determined for an E1 antigenic site (^314^TGHRMAWDMMM^324^) in complex with human nAb IGH526^28^,^29^. (n.b. residue range is indicated by the superscripts hereafter). The crystal structure (PDB ID 4N0Y) revealed an 11-aa helical peptide bound to a hydrophobic groove formed by the complementarity determining regions of the heavy chain (HCDRs) and light chain (LCDRs) of IGH526 (Fig. S2). The phenyl rings of a tyrosine (Y100E) and a phenylalanine (F98) in HCDR3 interact with a histidine (H316) and the aliphatic portion of an arginine (R317) in the E1 epitope, respectively, with additional stabilizing contacts provided by the base of LCDR3 (W91 and N95) and the last residue (W50) of framework 2 (FW2). Based on the structural and functional information, we hypothesized that a scaffold that can present this E1 helical epitope in the IGH526-bound conformation may elicit E1-specific nAbs, and furthermore, a β-sheet-rich scaffold environment surrounding the grafted E1 helical epitope may enhance the specificity of elicited antibody response as antibodies often employ different types of interaction to recognize epitopes of different secondary structures^45^.

**Figure 2:**
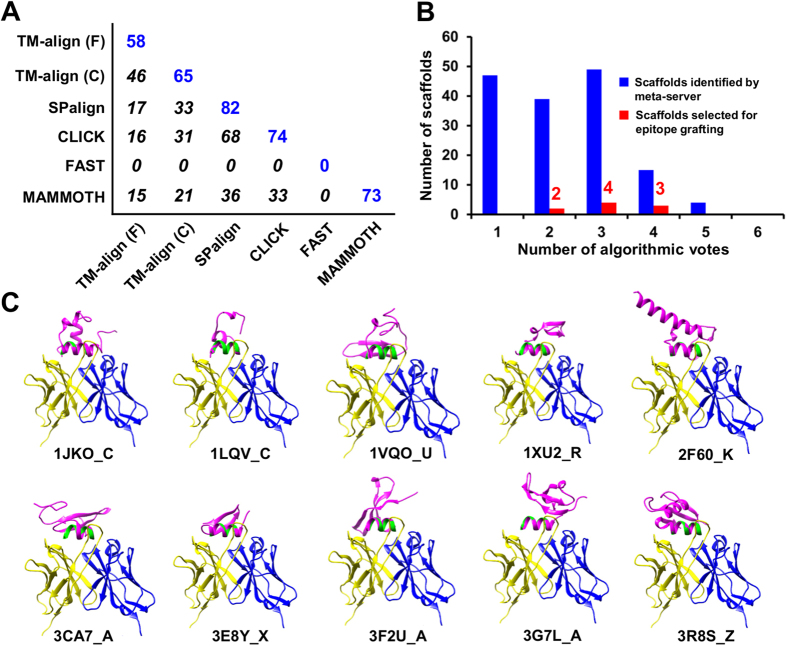
Computational design of epitope scaffolds for HCV E1 antigenic site 314–324. (**A**) Coverage matrix of scaffolds identified by six structural alignment algorithms used in the scaffolding meta-server. The number of scaffolds identified by each individual algorithm is shown in blue and the overlap between two algorithms is shown in *italic*. (**B**) Consensus analysis of the scaffolds identified by the scaffolding meta-server after filtering (blue) and manually selected for epitope grafting (red). The number of scaffolds is plotted as a function of the number of algorithms (votes) by which a scaffold is identified. A “cysteine knot” (PDB ID: 3CA7) is included to render a total of 10 scaffolds for epitope grafting, energy evaluation and explicit-water MD simulation (Figs S4–S6). (**C**) Ten protein scaffolds identified by the scaffolding meta-server and manual selection are superimposed onto the E1 epitope in complex with the human neutralizing antibody IGH526. All protein structures are shown as a ribbon model with the scaffold colored in magenta, epitope in green.

Collectively, the meta-server identified 704 non-redundant scaffolds, using input parameters consisting of a protein size of 30–60 aa, an epitope matching length of 10–11 aa, and a C_α_ RMSD cutoff of 2.0 Å. After removing scaffolds with a clash score over 5.0 with the docked IGH526, 154 candidates remained. We then calculated the number of scaffolds contributed by each algorithm and the overlap between any two algorithms (Fig. 2A). Similar to the 2F5 case, we observed an improved coverage of scaffold search. Among the six tested algorithms, SPalign appeared to be the best performer by providing 82 scaffolds, while the two versions of TM-align yielded 77 non-redundant scaffolds, accounting for 50% of the group. Surprisingly, FAST did not find any scaffolds, indicating that this algorithm may be less advantageous for short helices than for full-length proteins with distinctive structural features. We next ranked the scaffolds according to the count of algorithms (“votes”) by which they were identified (Figs 1, 2B). As expected, fewer scaffolds were found as the count increases, with 49 by three, 15 by four, 4 by five, and none by all six algorithms. After manual inspection, a total of 9 scaffolds were selected from the voting groups with 2, 3 and 4 counts (Fig. 2B). Criteria such as scaffold size, topology, flexibility, and structural environment of the epitope were major considerations in the selection^45^. We also included a cysteine knot, 3CA7_A, which has been used to scaffold the HIV-1 V3 epitope^42^ (n.b. a scaffold is denoted by the PDB ID followed by chain ID hereafter, which specifies a unique entry in the non-redundant protein database compiled by PISCES^54^, see Methods). Together, ten scaffolds were advanced to the design stage (Table 1, Table S1 and Fig. 2C), with 8–11 residues matched to the epitope by a C_α_ RMSD of 1.6 Å or lower and an epitope exposure ratio of 0.55 or greater in the scaffold context, except for 1JKO_C.

A key requirement for epitope grafting is to maintain epitope-antibody interactions and to minimize unfavorable interactions between the graft and the scaffold environment. As indicated by the sequence alignment of epitope-matching regions (Fig. S3), on average 7–8 critical residues in the E1 antigenic site were grafted onto a scaffold by superimposing the backbone atoms (N, C_α_ and C). Up to four mutations were introduced to avoid the scaffold side chains occluding antibody access or affecting protein stability due to exposed hydrophobic patches or buried charges (Fig. 2C). The flexible N- or C-terminus that may interfere with IGH526 binding was also truncated. The designed epitope scaffolds, indicated by the postfix “_ES” hereafter, were then evaluated in the presence of IGH526 using two residue-based local quality functions, a statistical potential DFIRE^55^, and a tabulated soft-core van der Waals (vdW) function (Fig. S4). Both functions have been used to detect local structural errors in homology models^56^. For these antigens, the local DFIRE score ranges from −1.41 to 4.20 with an average score of 0.75 and, in most cases, is below the 2.0 cutoff except for two regions (residues 5–7 and 22–24) in 3CA7_A_ES. The local vdW score, however, reported potential clashes between the grafted epitope residues and IGH526 or, in some cases, the scaffold (Fig. S4). These clashes could be readily removed by side-chain remodeling and energy minimization (see Methods), suggesting they were likely caused by rigid-body grafting rather than structural errors. MD simulations were performed to study the dynamics of epitope scaffolds in solution. The average C_α_ RMSD showed different levels of conformational differences relative to the initial model, ranging from 0.83 to 3.72 Å (Fig. S5). This global structural mobility was confirmed by the C_α_ RMS fluctuation (C_α_ RMSF) calculated from the conformational ensemble generated for each designed antigen (Fig. S6). The grafted epitope appeared to remain stable relative to the scaffold, showing an average C_α_ RMSF of 0.90 Å, compared to 1.81 Å for the whole protein.

### Experimental validation and case analysis of the E1 epitope scaffolds

**Figure 3:**
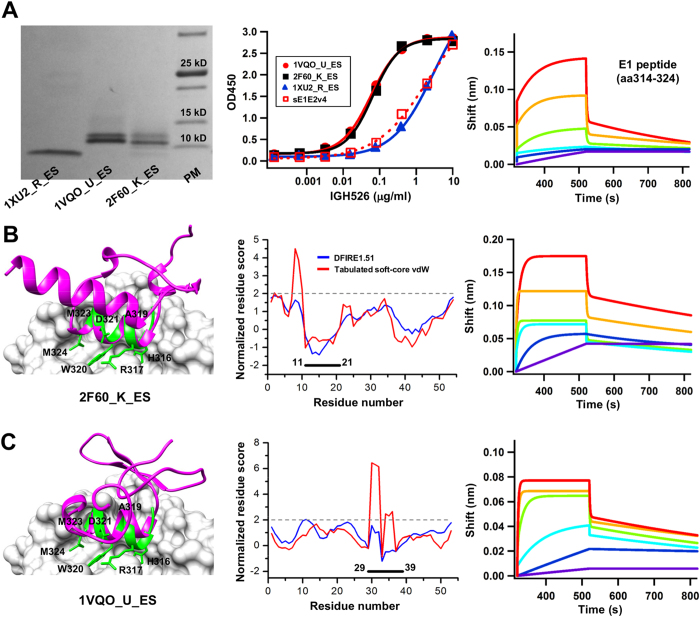
Experimental validation and case analysis of the E1 epitope scaffolds. (**A**) SDS PAGE analysis of three expressed epitope scaffolds containing the E1 antigenic site (314–324) under reducing conditions, followed by Coomassie blue staining (left), ELISA analysis of antigen binding to the neutralizing antibody IGH526 (middle), and Octet measurement of the E1 epitope-containing peptide (right). (**B,C**) Structural modeling (left), local quality assessment (middle), and Octet kinetics measurement (right) of the E1 epitope scaffolds designed based on (**B**) 2F60_K, and (**C**) 1VQO_U. The epitope scaffold model (magenta) is superimposed onto the epitope (green) in complex with IGH526 (surface). The side chains of the epitope residues grafted onto the scaffolds are shown in stick model and labeled. The normalized residue-based scores calculated from DFIRE (blue) and a tabulated soft-core vdW energy function (red) are plotted as a function of the residue number. The sensorgrams from the Octet RED96 system show the binding of E1 peptide and epitope scaffolds to IGH526 using an antigen titration series of six starting at a maximum concentration of 100 μg/ml and 50 μg/ml with two- and four-fold dilutions, respectively. The antibody concentration was fixed at 5 μg/ml.

His-tagged epitope scaffolds were expressed transiently in HEK293F cells, purified with size exclusion chromatography (SEC), and analyzed by reducing sodium dodecyl sulfate-polyacrylamide gel electrophoresis (SDS-PAGE). Three epitope scaffolds, 1VQO_U_ES, 1XU2_R_ES and 2F60_K_ES, showed expression on SDS-PAGE (Fig. 3A, left). The antigenic properties were assessed by enzyme-linked immunosorbent assay (ELISA), which showed more pronounced IGH526 binding for 2F60_K_ES and 1VQO_U_ES than for 1XU2_R_ES (Fig. 3A, middle), using a soluble E1E2^57^ as a control. Overall, the results indicate that the E1 antigenic site presented by these protein scaffolds can be recognized by antibody IGH526, for which the E1 peptide displayed slow-on/fast-off binding kinetics on Octet (Fig. 3A, right).

2F60_K is the binding domain (E3BD) of human dihydrolipoamide dehydrogenase (E3) binding protein (E3BP)^58^. Crystal structures revealed a rigid-body association of E3BD and E3, with a C_α_ RMSD of 0.7 Å between the bound and unbound forms of E3BD. Given a C_α_ RMSD of 0.28 Å between the N-terminal region (11–21) and the E1 antigenic site (Table 1), 2F60_K appeared to be an ideal scaffold to display this epitope (Fig. 3B, left). We transplanted E1 residues ^316^HR^317^, ^319^AWD^321^, and ^323^MM^324^ onto 2F60_K, and shortened the C-terminus (55–60) to reduce non-specific antibody response. We mutated the scaffold residues ^8^RF^9^ to alanine to avoid clashes with IGH526, and added a K40A mutation to reduce clashes with the grafted E1 R317 (Fig. 3B, left). The double mutation led to a more favorite DFIRE score, although the vdW score was still two-fold above the threshold (Fig. 3B, middle). In MD simulations, the grafted epitope showed an average C_α_ RMSF of 2.0 Å, compared to 2.7 Å for the whole protein. Using Octet, we quantified the IGH526 binding kinetics of 2F60_K_ES (Fig. 3B, right), which showed a faster on-rate than that of the E1 peptide (Fig. 3A, right).

1VQO_U is a 50S ribosomal protein (L24E) found in the large ribosomal subunit^59^ (Fig. 3C, left). We grafted E1 residues ^316^HR^317^, ^319^AWD^321^, and ^323^MM^324^ onto 1VQO_U, and introduced a double mutation T16G/F18A to minimize scaffold-antibody clashes. We also mutated the scaffold R41 to alanine to minimize clashes with the grafted E1 W320 and IGH526. We observed a similar pattern of local quality scores, with DFIRE indicating near-native contacts while vdW function suggesting potential clashes for residues 30–32, two of which (^316^HR^317^) were grafted from the E1 epitope (Fig. 3C, middle). MD simulations suggested that the grafted epitope would remain stable in the scaffold context, with an average C_α_ RMSF of 2.0 Å, compared to 2.3 Å for the whole protein. When tested for IGH526 binding using the Octet, 1VQO_U_ES showed a faster on-rate than 2F60_K_ES and the E1 peptide (Fig. 3C, right).

Taken together, the computational design coupled with experimental screening resulted in two antibody-reactive E1 epitope scaffolds with modest affinity and similar kinetics. Most scaffolds are components of large molecular complexes, explaining the difficulty in expressing these scaffold antigens as monomers (Fig. 3A, left). We also noted that the protein size cutoff (≤60 aa) might have excluded some larger scaffolds that are suitable for presentation of this epitope. Nevertheless, our results demonstrated that the E1 antigenic site can be displayed on heterologous protein scaffolds in a nAb-bound conformation.

### Epitope scaffold design for the E2 antigenic site 412–423

The E2 glycoprotein is a major target for neutralizing antibodies and is responsible for facilitating cell entry by interacting with its receptor CD81^60^. Several cross-genotype neutralizing antibodies recognizing E2 epitopes have been identified^23^,^24^,^25^. Crystal structures of an E2 antigenic site (^412^QLINTNGSWHIN^423^) in complex with murine antibody AP33^26^,^30^,^33^ and human antibody HCV1^31^ revealed a 12-aa epitope in the E2 N-terminus, which adopted a β-hairpin conformation with hydrophilic and hydrophobic faces on the opposing sides. Both antibodies recognize the CD81-receptor binding site (CD81bs) and neutralize HCV by blocking CD81 association. Remarkably, two independent antibody maturation pathways have arrived at a similar solution to engage the hydrophobic face of this epitope^30^,^31^. We hypothesized that a protein scaffold with an exposed β-hairpin resembling the structure of E2 antigenic site can be used to present this epitope in AP33- or HCV1-bound conformation and elicit epitope-specific neutralizing antibodies.

**Figure 4:**
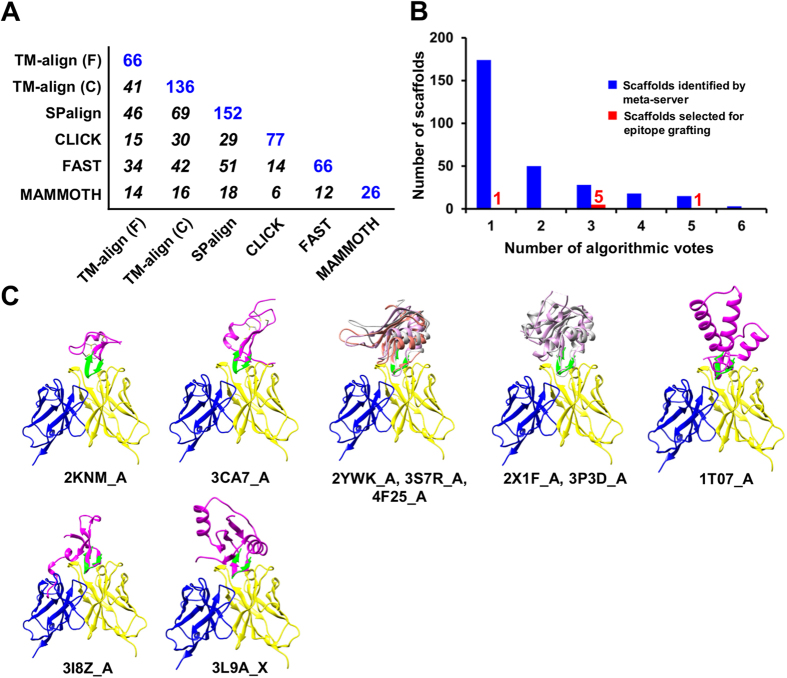
Computational design of epitope scaffolds for HCV E2 antigenic site 412–423. (**A**) Coverage of scaffolds identified by six structural alignment algorithms used in the scaffolding meta-server, with the number of scaffolds identified by each individual algorithm shown in blue and the overlap between two algorithms in *italic*. (**B**) Consensus analysis of the scaffolds identified by the scaffolding meta-server after filtering (blue) and manually selected for epitope grafting (red). The number of scaffolds is plotted as a function of the number of algorithms (votes) by which a scaffold is identified. Two “cysteine knots” (PDB IDs: 2KNM and 3CA7) and another protein (PDB ID: 3I8Z) are included to render a total of 10 scaffolds for epitope grafting, energy evaluation and explicit-water MD simulation (Figs S8–S10). (**C**) Ten protein scaffolds identified by the scaffolding meta-server and manual selection are superimposed onto the E2 epitope in complex with the human neutralizing antibody HCV1. All protein structures are shown as a ribbon model with the scaffold colored in magenta, epitope in green, with an exception for two groups of structural homologs for which the scaffolds are colored in magenta, gray, and orange.

A total of 2,564 non-redundant scaffolds were identified by the scaffolding meta-server, using coordinates of the E2 epitope as a template and search criteria comprising a protein size of 40–100 aa, an epitope-match length of 10–12 aa, and a C_α_ RMSD of 2.0 Å or less. Nearly 89% of the scaffolds were removed in the clash filtering step. The coverage matrix of scaffold search was determined for the remaining 288 scaffolds (Fig. 4A). Among these scaffolds, SPalign contributed 152, while the two TM-align algorithms provided a combined set of 161, accounting for 56% of the scaffolds. The consensus analysis revealed a different pattern of distribution, with majority of the scaffolds identified by a single algorithm (Fig. 4B). Based on similar criteria^45^, we manually selected 5 and 1 scaffolds from the voting groups with 3 and 5 counts, respectively, and a top-ranking candidate from the voting group with one count (Figs 1, 4B). We also included 2KNM_A and 3CA7_A to investigate the utility of “cysteine knots” as scaffolds to present the E2 antigenic site, and 3I8Z_A from a previous design study based on the structural similarity between the E2 epitope and the HIV-1 V3 epitope^42^ (Table 2, Table S2, and Fig. 4C). Further analysis revealed three scaffold groups: two structural homolog groups consisting of 2YWK_A, 3S7R_A and 4F25_A, and of 2X1F_A and 3P3D_A, respectively, and a cysteine knot group of 2KNM_A and 3CA7_A (Fig. 4C).

Two grafting strategies were explored to design E2 epitope scaffolds: to graft the entire hairpin (413–422) onto a scaffold, as exemplified for the cysteine knot group, or to graft the hydrophobic face only, as for the other eight scaffolds (Fig. 4C). Of the 6–7 grafted E2 residues, a conserved *N*-linked glycosylation site was located in the turn of the E2 hairpin, ^417^NGS^419^ (Fig. S7). Other potential *N*-linked glycosylation sites in the scaffolds, such as N38 in 4F25_A_ES and N58 in 3L9A_X_ES, were mutated to glutamine. Up to three mutations were introduced to the non-graft regions to remove steric clashes and unfavorable interactions and to improve the protein stability. As indicated by the overall favorite DIFRE scores (Fig. S8), most E2 epitope-HCV1 interactions appeared to be preserved, although minor clashes due to rigid-body grafting were detected by the vdW function. In MD simulations, the E2 epitope scaffolds have undergone a more visible global conformational shift compared to their E1 counterparts, with the average C_α_ RMSD ranging from 1.5 to 3.9 Å (Fig. S9). Despite the global shift, the epitope scaffolds showed less internal variation, with a C_α_ RMSF of 0.6 Å for the graft and a C_α_ RMSF of 1.4Å for the whole protein, respectively (Fig. S10).

### Experimental validation and case analysis of the E2 epitope scaffolds

**Figure 5:**
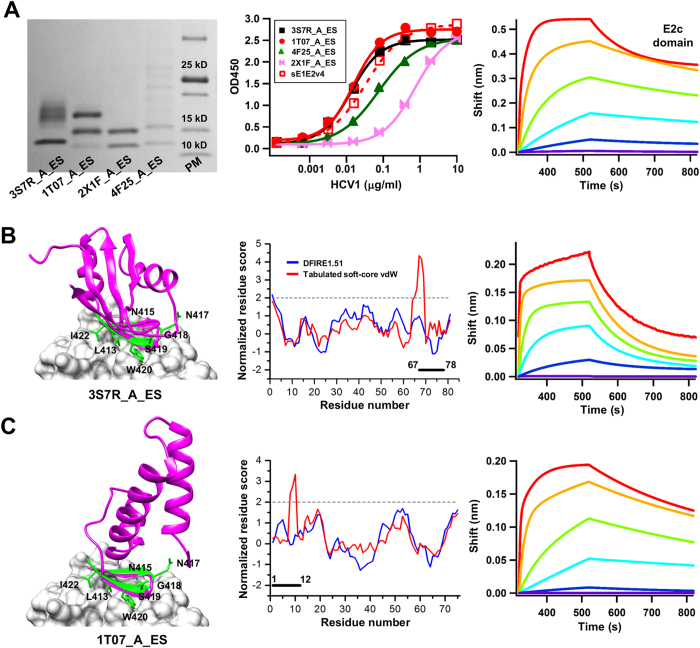
Experimental validation and case analysis of the E2 epitope scaffolds. (**A**) SDS PAGE analysis of five expressed epitope scaffolds containing the E2 antigenic site (412–423) under reducing conditions followed by Coomassie blue staining (left), ELISA analysis of E2 antigen binding to the human neutralizing antibody HCV1 (middle), and Octet measurement of the E2c domain (right). (**B,C**) Structural modeling (left), quality assessment (middle) and Octet measurements (right) of E2 epitope scaffolds designed based on (**B**) 3S7R_A, and (**C**) 1T07_A. The epitope scaffold model (magenta) is superimposed onto the epitope (green) in complex with HCV1 (surface). The side chains of the epitope residues grafted onto the scaffolds are shown in stick model and labeled. The normalized residue-based scores calculated from DFIRE (blue) and a tabulated soft-core vdW energy function (red) are plotted as a function of the residue number. The sensorgrams from the Octet RED96 system show the binding of E2c domain and E2 epitope scaffolds to HCV1 using an antigen titration series of six starting at a maximum concentration of 100 μg/ml and 25 μg/ml with four-fold dilutions, respectively. The antibody concentration was fixed at 5 μg/ml.

The E2 epitope scaffolds were expressed transiently in HEK293F cells, SEC purified, and assessed by reducing SDS-PAGE. Four designs derived from the scaffolds 4F25_A, 1T07_A, 3S7R_A, and 2X1F_A showed detectable expression on SDS PAGE, with multiple bands corresponding to different glycoforms of N417 (and an extra *N*-linked glycan in 1T07_A_ES) (Fig. 5A, left). These epitope scaffolds exhibited different HCV1 binding profiles in ELISA analysis, with 1T07_A_ES being the best performer (Fig. 5A, middle). 2KNM_A_ES, an epitope scaffold derived from a cysteine knot, bound to HCV1 at a similar level to 3S7R_A_ES in ELISA, but exhibited little expression on SDS PAGE. In summary, three scaffold groups each contributed at least one antigenic epitope scaffold, with 1T07_A_ES derived from a distinct fold family. The HCV1 binding was assessed for the E2c domain using the Octet, showing a fast-one/slow-off kinetics (Fig. 5A, right) in contrast to the kinetics observed for the E1 peptide (Fig. 3A, right).

Scaffolds 2YWK_A, 3S7R_A and 4F25_A are a group of RNA-binding proteins^61^ with an intra-group C_α_ RMSD of ~1.7 Å. Both 3S7R_A_ES and 4F25_A_ES bound to HCV1 with a similar binding profile by ELISA (Fig. 5A, middle). For scaffold 3S7R_A, we grafted epitope residues L^413^, N^415^, N^417^, ^419^SW^420^, and I^422^ to the scaffold, and mutated Q66 and K67 to alanine and glycine, respectively, to remove potential clashes with the antibody (Fig. 5B, left). Although the vdW score indicated steric clashes between ^65^DAGLH^69^ and HCV1, we observed a rather reasonable DFIRE score for this region (Fig. 5B, middle). Note that the highest-scoring residues ^67^GLH^69^ have extended into the region that anchors the β-hairpin epitope (Fig. S7). As stated previously, the high vdW scores were likely caused by rigid-body grafting rather than actual structural errors. When tested for the HCV1 binding, 3S7R_A_ES showed a less favorable binding kinetics than the E2c domain (Fig. 5B, right). For scaffold 1T07_A, a conserved protein from *Pseudomonas aeruginosa* with unidentified function, we grafted epitope residues L^413^, N^415^, ^417^NGSW^420^ and I^422^, and truncated the flexible N-terminus by four residues (Fig. 5C, left). Although the vdW function indicated some clashes between ^8^SWL^10^ and antibody HCV1, DFIRE showed a favorite local score for this region, indicating an acceptable quality of the designed interface (Fig. 5C, middle). In MD simulations, the grafted E2 epitope centered at the turn (residues 6–8) appeared to be more mobile relative to the scaffold, with a C_α_ RMSF of 2–4 Å, but relatively rigid within the region, with a local C_α_ RMSF of 0.5 Å (Fig. S10). For 1T07_A_ES, the Octet data showed slow-on/slow-off kinetics, indicative of a more desirable antigenic profile (Fig. 5C, right).

Overall, our results indicate that the E2 antigenic site is more amenable to the scaffolding approach, as half of the designed epitope scaffolds were reactive to antibody HCV1. Our results are also reminiscent of a previous scaffolding study where similar success rates were observed, with 55% for a hairpin-like epitope and 14% for a helical epitope, respectively^42^. The two best performers by ELISA, 3S7R_A_ES and 1T07_A_ES, showed different kinetics as determined by Octet, indicating that such biophysical measurements are critical in a quantitative assessment and comparison of scaffold antigen designs of the same epitope.

### Multivalent scaffolding

With highly symmetric, repetitive antigen display on the surface, virus-like particles (VLPs) can induce potent immune response and have been used as vaccines against cognate viruses or vaccine platforms to carry foreign antigens^62^,^63^. It has been demonstrated that a minimum of 20–25 epitopes spaced by 5–10 nm are sufficient for effective B-cell activation^66^. Therefore, nanoparticles with similar molecular traits to that of VLPs may be used as multivalent scaffolds to present an epitope scaffold, providing useful tools to probe the immune response to the presented epitope. For example, the 60-mer lumazine synthase particle from *Aquifex aeolicus* has been used to display an engineered HIV-1 gp120 outer domain to target a specific antibody germline VH gene^67^. The 24-mer ferritin particle from *Helicobacter pylori* was used to present influenza hemagglutinins (HAs) and elicited H1N1-neutralzing antibodies in ferrets^68^. Ferritin was also used as a carrier of scaffold antigen designs of the HIV-1 V3 epitope^42^, suggesting that ferritin is a robust particle platform for designing and testing multivalent antigens.

**Figure 6:**
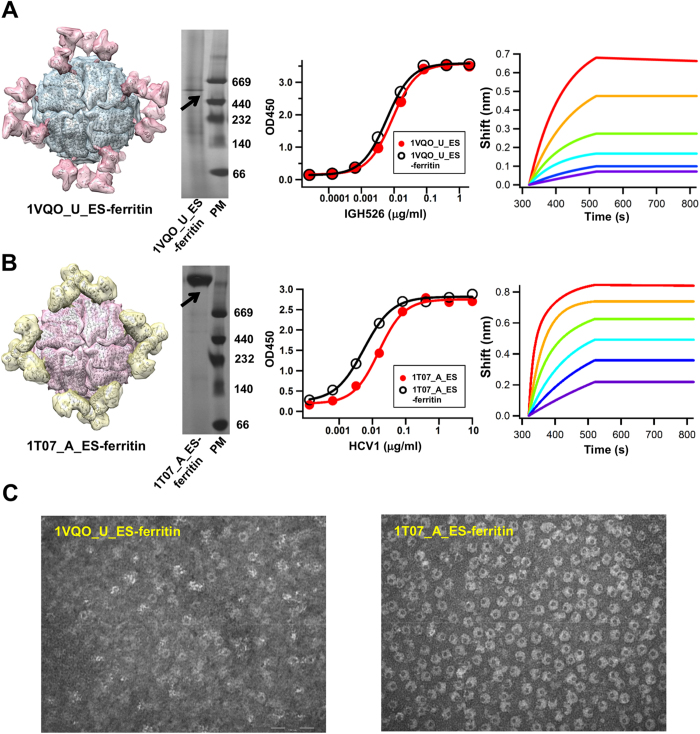
Design and characterization of nanoparticle antigens. For E1 epitope scaffold 1VQO_U_ES (**A**) and E2 epitope scaffold 1T07_A_ES (**B**), the modeled ferritin nanoparticle displaying twenty-four copies of the antigen (left), blue native (BN) PAGE of the nanoparticle (middle left), ELISA binding of individual epitope scaffold and its particle form to the respective antibody (middle right), and Octet kinetics measurement of the nanoparticle (right) are shown. The molecular surface of the nanoparticle model is color-coded differently for ferritin and attached epitope scaffold. Coomassie blue staining was used after BN PAGE. IGH526 and HCV1 were used in ELISA and Octet experiments to test the antibody binding of E1 and E2 nanoparticle antigens, respectively. The sensorgrams show the nanoparticle-antibody binding using an antigen titration series of six starting at the maximum concentration of 10 μg/ml (calculated based on a single subunit) with two-fold dilutions. The antibody concentration was fixed at 5 μg/ml. (**C**) Negative-stain electron microscopy (EM) images are shown for 1VQO_U_ES-ferritin (left) and E2 epitope scaffold 1T07_A_ES-ferritin (right) at a magnification of 245K × for both particle samples.

We first investigated whether ferritin particle can be used to present an E1 epitope scaffold, 1VQO_U_ES. The C-terminus of this antigen was fused to the N-terminus of a ferritin subunit (Asp5) with a 5-aa flexible linker. If such fusion protein is capable of assembling into a particle, 24 copies of the E1 epitope scaffold will be displayed on the surface (Fig. 6A, left). Indeed, blue native (BN) PAGE showed a band at the molecular weight of ~624 kD, consistent with properly formed 24-mer particles (Fig. 6A, middle left). Compared to the monomeric epitope scaffold, the nanoparticle only showed a slight shift of IGH526 binding profile by ELISA (Fig. 6A, middle right), but a significantly improved off-rate as indicated by Octet (Fig. 6A, right). The immeasurable dissociation is likely due to increased avidity (with 24 binding sites exposed on the particle surface) and consistent with a previous study^42^.

We then extended the ferritin particle design to the E2 epitope scaffolds based on a similar fusion strategy. The chimeric protein showed comparable if not higher expression than the epitope scaffold alone, suggesting that a robust particle platform like ferritin can also be used to improve the expression of a designed antigen. Structural modeling of the 1T07_A_ES-ferritin particle (Fig. 6B, left) indicated that this E2 epitope scaffold would form propeller-shape trimers on the particle surface, with exposed E2 epitopes pointing away from the 3-fold axes. A single band was seen on the BN-PAGE gel (Fig. 6B, middle left), confirming proper assembly of antigen-carrying ferritin particles. Compared to 1VQO_U_ES, we observed a more notable shift of the HCV1 binding profile in ELISA (Fig. 6B, middle right). Remarkably, the 1T07_A_ES nanoparticle exhibited a lower than picomolar affinity on Octet, with plateaued dissociation curves under all tested concentrations, suggesting a strong avidity effect (Fig. 6B, right). A similar characterization was performed for 2KNM_A_ES-ferritin (Fig. S11), indicating that multiple E2 epitope scaffolds are suitable for particulate presentation.

We also validated the particle assembly of 1VQO_U_ES-ferritin (Fig. 6C, left) and 1T07_A_ES-ferritin (Fig. 6C, right) by negative-stain electron microscopy (EM), which showed results in agreement with their antigenic profiles. 1VQO_U_ES-ferritin particles were visible with moderate density and purity, compared to the remarkable numbers of homogeneous particles observed for 1T07_A_ES-ferritin. The “layered” structure of 1T07_A_ES-ferritin particles (Fig. 6C, right) is likely attributed to the 48 *N*-linked glycans displayed on the surface (2 × 24 subunits). Taken together, our results demonstrated that self-assembling nanoparticles such as ferritin can be used as a multivalent scaffold to present a neutralizing epitope in high density. Such multi-scale scaffolding strategy may prove to be useful in designing immunogens to elicit a robust antibody response to a target epitope. The HCV nanoparticles designed in this study will be further tested *in vivo* to examine this possibility.

## Discussion

Vaccines provide cost-effective tools to improve public health and to prevent infectious diseases. Despite great success, broadly effective vaccines have not been developed for viral pathogens such as HIV-1, HCV, and influenza^69^. Protein engineering has been applied to the development of novel diagnostic and therapeutic agents, for which cysteine knots^70^,^71^, designed ankyrin repeat proteins (DARPins)^72^,^73^, and other unique protein folds have been used as scaffolds to display a functional site. It was only recently that the concept of scaffold was extended to epitope vaccine design by grafting an epitope of interest onto heterologous protein scaffolds. The scaffolding approach has proved to be useful for epitope vaccine design, with case studies reported for HIV-1^40^,^41^,^42^,^46^,^47^, Flu^74^, and RSV^43^,^44^. For HCV, a number of cross-neutralizing epitopes have been identified and structurally characterized^23^,^24^,^25^,^30^,^31^,^32^,^33^,^35^, but not yet subjected to vaccine design by the scaffolding approach. Scaffolding-based epitope vaccine design builds upon the assumption that the available protein database contains scaffolds that can structurally accommodate an epitope of interest and a scaffolding algorithm can identify such needles from a large haystack. Since most scaffolding studies have been conducted using a single structural alignment algorithm, a large repository of other potential techniques was unexamined. In this study, we borrowed the concept of meta-server^75^,^76^ to exploit the combined strengths of six structural alignment algorithms for scaffold search. For both HCV antigenic sites studied, the scaffolding meta-server consistently provided a more complete set of scaffolds for epitope scaffold design. Biochemical and antigenic characterization then played a critical role in the screening of designed antigens (Figs 3 and 5, and Figs S12 and S13). We also attempted to bridge the gap between epitope scaffolding and the VLP vaccine concept by utilizing ferritin nanoparticle as a multivalent scaffold to present an antigenic epitope scaffold. With a diameter of ~12 nm and 24 fusion sites on the surface, ferritin particle can serve as a model system to experiment with various VLP designs without involving complex purification and polishing process typical of the VLP approaches^77^. Other self-assembling nanoparticles, especially those of microbial origin, may also be exploited for this purpose. To our knowledge, this study represents the first attempt to design epitope vaccines for HCV by a scaffolding approach, and a set of novel immunogen candidates is now awaiting *in vivo* trials.

## Methods

### Protein structure database

A protein structure database was compiled for scaffold search as previously described^42^. Briefly, the PISCES server^54^ was used to generate a representative, non-homologous set of Protein Databank (PDB) entries at the 90% identity level, with a resolution cutoff of 3.0 Å, and an R-factor cutoff of 1.0. Of note, these parameters were selected to increase the coverage of the resulting database. As of February, 2013, the PISCES server yielded a list of 23,576 peptide chains after screening using the above parameters, with the chemically modified residues corrected and C_α_-only PDB files removed. Each entry in the PISCES database contains a PDB ID and a chain ID, which provide a unique identifier for scaffold search.

### A semi-automated pipeline for epitope scaffold design

Epitope scaffold design is a critical component of the epitope vaccine strategy^44^. In this study, we have developed a semi-automated pipeline for epitope scaffold design (Fig. 1). Given an antibody-antigen complex with a well-defined epitope, this pipeline consists of five steps: (1) scaffold search and filtering using a scaffolding meta-server, (2) consensus analysis to determine the coverage of scaffold search and to prioritize the scaffold list, (3) scaffold selection based on a set of structural criteria, (4) epitope grafting by rigid-body fitting, and (5) energy evaluation using residue-based scoring functions and structure optimization. The output is a list of epitope scaffold models.

In step one, a scaffolding meta-server is used to identify diverse scaffolds. In this method, six structural alignment algorithms including two versions of TM-align^50^, SPalign^51^, CLICK^52^, FAST^53^, and MAMMOTH^49^ are combined to search the PISCES database. These algorithms are based on diverse scoring functions and search schemes: TM-align generates structural alignment by optimizing TM-score in a dynamic programming (DP) scheme; SPalign optimizes a size-independent objective function and provides both SP-score and TM-score as output; CLICK is a graph theory algorithm that takes into account structural information such as C_α_ coordinates, secondary structure, solvent accessibility and residue depth; FAST relies on heuristic search to approach the optimal structural alignment; MAMMOTH maximizes the overlap between two protein structures using a sequence-independent heuristic search scheme. The coordinates of the target epitope are used as input to six structural alignment algorithms for scaffold search. The output from each algorithm is processed on the fly, resulting in a list of PDB entries with detailed parameters such as protein size, number of matched residues, C_α_ RMSD, and epitope exposure ratio (epitope surface in the context of a scaffold versus solvent-exposed peptide). In the filtering step, scaffolds with a clash score of 5.0 or greater in the docked antibody-scaffold complex are removed. The clash score is calculated for all heavy atoms of the antibody-scaffold interface within 1.0 Å using the sum of 1/*r* (*r*: the atomic distance).

In step two, protein scaffolds identified by the meta-server are subjected to a consensus analysis to calculate the “coverage matrix”. Specifically, the number of scaffolds contributed by each algorithm and the pairwise overlap are determined (Fig. S1, Figs 2A, 4A). This matrix provides a quantitative measure of scaffold search coverage. Each scaffold is assigned to a voting group based on the number of algorithms by which the scaffold is identified. The voting count provides a basis for consensus-based scaffold selection.

In step three, the top-ranking scaffolds are manually inspected based on criteria such as protein size, topology, backbone flexibility, and structural environment of the epitope-matching region. Briefly, small, inherently flexible scaffolds with the epitope-matching region situated in a different secondary structural environment (e.g. a helical epitope in a β-rich protein) or separated from the surrounding structures with well-defined boundaries are preferred. The rationales for these selection criteria have been explained in detail^45^.

In step four, transplantation of epitope residues is performed by rigid-body fitting of the backbone atoms (N, C_α_ and C). After epitope grafting, scaffold residues with side chains within a 6.5 Å distance to the grafted epitope side chains or antibody side chains are mutated to eliminate potential steric clashes. Exposed hydrophobic residues or buried charges in the scaffold are also mutated to improve the solubility and stability of the designed antigen.

In step five, two residue-based scoring functions are used to assess the structural quality of the designed epitope scaffold antigen in the presence of the antibody after rigid-body docking. DFIRE has been used to distinguish the native structure from decoy models^55^, predict protein-protein interface^78^, and refine protein models with or without experimental constraints^56^,^79^,^80^,^81^. The tabulated soft-core vdW function has been applied to homology model refinement^56^. In structural quality assessment, if the normalized residue score is above 2.0, the side chains involved will be subjected to an in-house side-chain modeling program that combines an XYZ side-chain rotamer library^81^, DFIRE^54^, and a FASTER search algorithm^83^, to obtain the optimal conformation. A 50-step energy minimization using TINKER^84^ and OPLS all-atom force field^85^ is performed to relax the epitope scaffold structure. Side-chain modeling coupled with a short energy minimization has been found to effectively reduce the clash score.

### MD simulation of epitope-scaffolds

The GROMACS 3.3.3 package^86^,^87^ was used to perform MD simulation on the designed epitope scaffolds. The GROMOS96 43a1 force field was used. The protonation state of ionizable amino acids was set for a pH of 7.0, with counterions added to neutralize the system. Each epitope scaffold was solvated in a rectangular box using the SPC water model. The minimal distance between the protein and the wall of the unit cell was set to be 10 Å. A non-bound pair-list with cutoff of 9.0 Å was used, and the pair-list was updated every five time steps. A twin-range method was used to calculate the van der Waals interactions. Interactions within the short-range cutoff (9.0 Å) were updated every step, while interactions with the long-range cutoff (14 Å) were updated every five steps together with the pair-list. The electrostatic interaction within the short-range cutoff (9.0 Å) was calculated with Columbic function while those beyond the cutoff were treated with the particle mesh Ewald method (PME)^88^. LINCS algorithm^89^ and SETTLE algorithm^90^ were used to constrain covalent bonds in the proteins and the geometry of the water molecules, respectively. A time step of two femtoseconds was used. The protein/ion/water system was first energy minimized and then equilibrated in a 100-picosecond MD simulation with positional restraints on the heavy atoms of the protein. The production run (5 × 5 nanoseconds) was performed at a temperature of 300 K and a constraint pressure of one bar by coupling to an external heat and an isotropic pressure bath^91^. Snapshots were stored every one picosecond, resulting in a total of 25,000 conformations for each epitope scaffold, which were used in the MD trajectory analysis.

### Expression and purification of designed HCV antigens

The designed HCV antigens were transiently expressed in HEK293F cells (Life Technologies, CA). Briefly, 293F cells were thawed and incubated with FreeStyle^TM^ 293 Expression Medium (Life Technologies, CA) in the Shaker incubator at 37 °C, with 120 rpm and 8% CO_2_. When the cells reached a density of 2.0 × 10^6^/ml, expression medium was added to reduce cell density to 1.0 × 10^6^/ml for transfection with polyethyleneimine (PEI) (Polysciences, Inc). 1000 μg plasmid DNA in 25 ml of Opti-MEM transfection medium (Life Technologies, CA) was mixed with 4 ml of PEI-Max (1.0 mg/ml) in 25 ml of Opti-MEM. After incubation for 25 min, the DNA-PEI-Max complex was added to 1 L 293 F cells. Culture supernatants were harvested five days after transfection, clarified by centrifugation at 3000 rpm for 10 min, and filtered using a 0.22 μm filters (Thermo Scientific). Proteins were purified from the supernatants using Ni Sepharose^TM^ excel column (GE Healthcare) and then further purified using size exclusion chromatography (SEC) on a Superdex 200 10/300 GL column (GE Healthcare). Protein concentrations were determined using UV_280_ absorbance with theoretical extinction coefficients.

### SDS PAGE and Blue Native (BN) PAGE

HCV antigens were analyzed using sodium dodecyl sulfate-polyacrylamide gel electrophoresis (SDS PAGE) and BN PAGE and stained using Coomassie blue. The protein samples were mixed with loading dye and loaded onto a 4–12% Bis-Tris NuPAGE gel (Life Technologies) or a 10% Tris-Glycine Gel (Bio-Rad). The SDS PAGE gels were run for 20 min at 250 V using the SDS running buffer (Bio-Rad). BN PAGE gels were run for 1.5 h at 200 V using NativePAGE^TM^ running buffer (Life Technologies) according to the manufacturer’s instructions.

### ELISA binding assays

Costar^TM^ 96-well assay plates (Corning) were coated with the HCV antigen overnight at 4 °C. The wells were washed once with PBS + 0.05% Tween 20, and then incubated with 150 μl of blocking buffer (PBS with 5% w/v dry milk) per well for 1 hour at room temperature (RT) followed by 5 times of washing in PBS + 0.05% Tween 20. 50 μl of HCV-specific antibodies in blocking buffer were added, with a maximum concentration of 2 μg/ml and a 5-fold dilution series, and incubated for 1 hour at RT. After washing 5 times in PBS + 0.05% Tween 20, the wells were incubated with 50 ul of Peroxidase-AffiniPure Goat Anti-Human IgG antibody (Jackson ImmunoResearch Laboratories, Inc) at 1:5000 in PBS + 0.05% Tween 20 per well for 1 h at RT. After washed 5 times in PBS + 0.05% Tween 20, the wells were developed using TMB at RT for 5 min and the reaction stopped with 180 mM HCl. The readout was measured at a wavelength of 450 nm.

### Octet binding assays

The kinetics of the HCV antigens binding to their respective antibodies was measured using an Octet Red96 instrument (fortéBio). All assays were performed with agitation set to 1000 rpm in fortéBIO 1 × kinetic buffer. The final volume for all the solutions was 200 μl/well. Assays were performed at 30 °C in solid black 96-well plates (Geiger Bio-One). 1 μg/ml of protein in 1 × kinetic buffer was used to load the HCV antibody IGH526 or HCV1 on the surface of anti-human Fc Capture Biosensors (AHC) for 300 s. Typical capture levels were between 0.5 and 1 nm and variability within a row of eight tips did not exceed 0.1 nm. A 60 s biosensor baseline step was applied prior to the analysis of the association of the antibody on the biosensor to the antigen in solution for 200 s. Two-fold concentration gradient of HCV antigen starting at 10 μg/ml was used in a titration series of six. The dissociation of the interaction was followed for 300 s. Correction of baseline drift was performed by subtracting the averaged shift recorded for a sensor loaded with HCV antibody but not incubated with HCV antigen, or a sensor without HCV antibody but incubated with HCV antigen. Octet data were processed by fortéBio’s data acquisition software v.8.1. Experimental data were fitted with the binding equations describing a 1:1 interaction. Local fitting of the data sets was performed to obtain the optimal results for different HCV antigens examined. The K_D_ value was determined using the estimated response at equilibrium for each antigen concentration rather than the *k*_on_ and *k*_dis_ values.

### Negative-stain electron microscopy (EM)

Copper grids (carbon coated, 400 mesh (Electron Microscopy Sciences, Hatfield PA)) were glow discharged and inverted on a 7 μl aliquot of sample for 3 minutes. Excess sample was removed and the grids immediately placed briefly on a droplet of double distilled water followed by 1% uranyl formate solution for 2 minutes. Excess stain was removed and the grid allowed to dry thoroughly. Grids were then examined on a Philips CM100 electron microscope (FEI, Hillsbrough OR) at 80 kv and images collected using a Megaview III ccd camera (Olympus Soft Imaging Solutions GmbH, Münster, Germany).

## Additional Information

**How to cite this article**: He, L. *et al.* Approaching rational epitope vaccine design for hepatitis C virus with meta-server and multivalent scaffolding. *Sci. Rep.* **5**, 12501; doi: 10.1038/srep12501 (2015).

## Supplementary Material

Supplementary Information

## Figures and Tables

**Table 1:** Protein scaffolds identified for HCV glycoprotein E1 antigenic site 314–324^a^.

#	PDB ID	Chain	Selection	N_res_	N_ali_	C_α_ RMSD (Å)	TM-score	SA ratio	Clash	Fitting	Votes
1	1JKO	C	Meta-server	46	10	0.41	0.32	0.39	0.00	TM-align (F)	2
2	1LQV	C	Meta-server	33	11	0.57	0.33	0.62	1.53	TM-align (F)	4
3	1VQO	U	Meta-server	53	10	1.59	N/A^c^	0.57	0.00	MAMMOTH	3
4	1XU2	R	Meta-server	36	11	1.24	0.26	0.82	1.11	TM-align (F)	3
5	2F60	K	Meta-server	60	11	0.28	0.31	0.56	1.05	TM-align (F)	4
6	3CA7^b^	A	Cysteine knot	50	8	0.25	0.26	0.55	2.69	TM-align (F)	−
7	3E8Y	X	Meta-server	30	10	0.24	0.42	0.63	4.67	TM-align (F)	2
8	3F2U	A	Meta-server	51	11	1.23	0.31	0.70	0.00	TM-align (F)	3
9	3G7L	A	Meta-server	55	10	0.16	0.30	0.67	1.99	TM-align (F)	3
10	3R8S	Z	Meta-server	58	11	0.49	0.30	0.57	0.00	TM-align (F)	4

^a^Listed items include scaffold index, PDB identifier, selection method, chain name, number of residues in the scaffold, number of scaffold residues aligned to the epitope, C_α_ RMSD of aligned residues, solvent accessibility ratio of the epitope-matching region in the scaffold context versus the epitope alone, scaffold-antibody clash score after docking the scaffold into the epitope-antibody complex, fitting algorithm used in the scaffold docking, and the number of algorithmic votes each scaffold received.

^b^Selected from the “cysteine knot” structural superfamily or a set of scaffolds identified for the HIV-1 epitope at the V3 base recognized by a broadly neutralizing antibody PGT128 (Ref. 41).

^c^TM-score was only calculated for the scaffolds identified and fitted by TM-align and SPalign, which provide TM-score as part of the output.

**Table 2:** Protein scaffolds identified for HCV glycoprotein E2 antigenic site 412–423^a^.

#	PDB ID	Chain	Selection	N_res_	N_ali_	C_α_ RMSD (Å)	TM-score	SA ratio	Clash	Fitting	Votes
1	2KNM^b^	A	Cysteine knot	30	2	0.02	N/A^c^	0.09	1.65	Manual fitting	−
2	3CA7^b^	A	Cysteine knot	50	6	0.61	N/A^c^	0.31	11.58	Manual fitting	−
3	2YWK	A	Meta-server	84	12	1.26	0.19	0.57	0.00	TM-align (C)	3
4	3S7R	A	Meta-server	81	12	1.48	0.20	0.56	0.00	TM-align (F)	3
5	4F25	A	Meta-server	81	10	1.10	N/A^c^	0.58	0.00	FAST	3
6	2X1F	A	Meta-server	94	12	1.43	0.18	0.72	0.00	TM-align (C)	3
7	3P3D	A	Meta-server	87	12	1.11	0.20	0.60	0.00	TM-align (C)	3
8	1T07	A	Meta-server	81	12	1.77	0.18	0.51	0.00	TM-align (C)	1
9	3I8Z^b^	A	PGT128 scaffold	50	12	0.70	0.32	0.71	36.78	TM-align (F)	−
10	3L9A	X	Meta-server	81	12	1.18	0.22	0.64	1.60	TM-align (F)	5

^a-c^See Table 1 for detailed explanation of listed items
