# Supplementary material for: Approaching rational epitope vaccine design for hepatitis C virus with meta-server and multivalent scaffolding
Source: Sci Rep. 2015 Aug 4;5:12501. doi: 10.1038/srep12501 (PMC4533164; doi:10.1038/srep12501)
Supplement: Supplementary Information [file srep12501-s1.pdf]

Supplementary Information (SI) for

Approaching rational epitope vaccine design for hepatitis C virus with meta-server and multivalent scaffolding

Linling He[1*], Yushao Cheng[1*], Leopold Kong[2,3,4], Parisa Azadnia[1], Erick Giang[1], Justin Kim[1], Malcolm R. Wood[5], Ian A. Wilson[2,3,4], Mansun Law[1§] & Jiang Zhu[1,2,4§]

[1] Department of Immunology and Microbial Science, The Scripps Research Institute, La Jolla, California 92037, USA.
[2] Department of Integrative Structural and Computational Biology, The Scripps Research Institute, La Jolla, California 92037, USA.
[3] International AIDS Vaccine Initiative, Neutralizing Antibody Center and the Collaboration for AIDS Vaccine Discovery.
[4] Center for HIV/AIDS Vaccine Immunology and Immunogen Discovery, The Scripps Research Institute, La Jolla, California 92037, USA.
[5] Core Microscopy Facility, The Scripps Research Institute, La Jolla, California 92037, USA.

This file includes:

Tables 1-2

Figures 1-13

Table S1. Design and sequence information of 10 epitope scaffolds presenting an E1 antigenic site (314-324) recognized by a murine antibody IGH526.[a]

ES name	PDB ID (Chain ID)	Epitope transplantation and mutations	Amino acid sequence
IGH526-ES1	1JKO (C)	delete1-9+E10A+Q11G+E12H+Q13R+S15A+R16W+E19M+K20M+delete45-46	AGHRIAWLLMMGHPRQQLAIIFGIGVSTLYRYFPA
IGH526-ES2	1LQV (C)	N2F+F4K+L5S+L8A+F24H+E25A+K28W+E29D+F31M+Q3M+delete33	AFSKSEEARHSSLERECIEEICDHAEAWDIMM
IGH526-ES3	1VQO (U)	T16G+F18A+K21G+S31H+K32R+E34A+N35W+N36D+D38M+L39M+R41A	RECDYCGTDIEPGTGGMAVHGDGATTHFCSHRCAWDAMMGAEARNLEWTDTAR
IGH526-ES4	1XU2 (R)	S23G+delete24-25+P26A+L28H+T29R+Q31A+R32W+Y33D+N35M+A36M	CSQNEYFDSLLHACIPCQLRCSGAPHRCAWDCMM
IGH526-ES5	2F60 (K)	R8A+F9A+P13H+A14R+R16A+N17W+I18D+E20M+K21M+K40A+delete55-60	EHIPGTLAARLSHRAAWDLMMHSLDASQGTATGPRGIFTAEDALKLVQLKQTGK
IGH526-ES6	3CA7 (A)	P8G+E9H+T10R+D12A+A13W+W14D+graft(CMM)+insert(GSG, connect17)+D19G+H21A	TFPTYKCGHRFAWDCMMGSGLNGAACFAVKIADLPVYSCECAIGFMGQRCEYKE
IGH526-ES7	3E8Y (X)	S4G+S5H+D6R+R8A+V9W+K10D+V12M+A13M+M14L	ACYGHRCAWDCMMLGFSSGKCINSKCKCYK
IGH526-ES8	3F2U (A)	R11K+E37D+C42G+P43H+D44R+I46A+A47W+E48D+L50M+Q51M	GEYVVEKVLDKRVVKGKVEYLLKWKGFSDEDNTWEPDENLDGHRLAWDFMM
IGH526-ES9	3G7L (A)	D8A+E46G+K47H+V48R+K50A+K51W+K53M+K54M	ADVYEVEAILADRVNKNGINEYYIKWAGYDWYDNTWEPEQNLFGAGHRLAWWMMR
IGH526-ES10	3R8S (Z)	K2G+K18A+T40G+P41H+A42R+R44A+R45W+M46D+N48M+A49M+E58G	AGTIKITQTRSAIGRLPAHKATLLGLGLRRIGHTVEREDGHRIAWDIMMVSFMVKVEG

[a] Listed items include epitope scaffold name, PDB ID (chain), brief description of epitope transplantation and mutations introduced in the computational design, and amino acid sequence of the epitope scaffold antigens.

Table S2. Design and sequence information of 10 epitope scaffolds presenting an E2 antigenic site (412-423) recognized by a human antibody HCV1.[a]

ES name	PDB ID (Chain ID)	Epitope transplantation and mutations	Amino acid sequence
HCV1-ES1	2KNM (A)	24-25->LINTNGSWHI+V9G+W10S+delete11	GIPCGESCGSPCISSAIGCSCKLINTNGSWHIVCYRN
HCV1-ES2	3CA7 (A)	delete1-4+Y5G+26-31->LINTNGSWHI	GKCPETFDAWYCLNDAHCFAVLINTNGSWHIVYSCECAIGFMGQRCEYKE
HCV1-ES3	2YWK (A)	L66A+N67G+G68L+R70N+Y72N+G73G+R74S+P75W+N77I	QEEADRTVFVGNLEARVREEILYELFLQAGPLTKVTICKDREGKPKSFGFVCFKHPESVSYAIALAGLINLNGSWIIVSGPSSG
HCV1-ES4	3S7R (A)	Q66A+K67G+E68L+R70N+D72N+G73G+R74S+V75W+D77I	NEEDAGKMFVGGLSWDTSKKDLKDYFTKFGEVVDCTIKMDPNTGRSRGFGFILFKDAASVEKVLDAGLHNLNGSWIIPKKA
HCV1-ES5	4F25 (A)	N38Q+E59A+N62G+G63L+L65N+N67N+D68G+R69S+K70W+F72I	SGNIFIKNLDKSIDNKALYDTFSAFGNILSCKVVCDEQGSKGYGFVHFETQEAAERAIAKMGLMNLNGSWVIVGRFKSRKE
HCV1-ES6	2X1F (A)	R62G+N63A+N65G+G66L+Q68N+G70N+S71G+R72S+F73W+K75I	PSRVVYLGSIPYDQTEEQILDLCSNVGPVINLKMMFDPQTGRSKGYAFIEFRDLESSASAVGALGLYNLNGSWLICGYSSNSDISGVSLEHHHH
HCV1-ES7	3P3D (A)	L73A+N75G+G76L+V78N+N80N+G81G+V82S+L83W+G85L	LAILVFGYPETMANQVIAYFQEFGTILEDFEVLRKPQAMTVGLQDRQFVPIFSGNSWTKITYDNPASAVDALAEGLANFNGSWLLVIPYTKDAVERLQ
HCV1-ES8	1T07 (A)	delete1-4+T6L+M8N+10-13->NG+E14S+E15W+P17I	RLVNCNGSWLIGLDRPPYPGAKGEDIYNNVSRKAWDEWQKHQTMLINERRLNMMNAEDRKFLQQEMDKFLSGEDY
HCV1-ES9	3I8Z (A)	K9L+I11N+K13N+R15S+V16W+Y18I+L39G+delete40-50	FAVESIEKLRNRNGSWEILVKWRGWSPKYNTWEPEENIG
HCV1-ES10	3L9A (X)	E11L+T13N+A15N+G16G+V17S+H18W+A20I+N58Q+H81G	MRDFFVITNSLYNFNGSWYIKGAVLHVSPTQKRAFWVIADQENFIKQVNKNIEYVEKQASPAFLQRIVEIYQVKFEGKNVG

[a] Listed items include epitope-scaffold name, PDB ID (chain), brief description of epitope transplantation and mutations introduced in the computational design, and amino acid sequence of the epitope scaffold antigens.

	TM-align (F)	TM-align (C)	SPalign	CLICK	FAST	MAMMOTH
**TM-align (F)**	**262**					
**TM-align (C)**	***181***	**539**				
**SPalign**	***108***	***202***	**331**			
**CLICK**	***71***	***113***	***103***	**74**		
**FAST**	***25***	***78***	***48***	***22***	**152**	
**MAMMOTH**	***79***	***133***	***148***	***64***	***36***	**273**

Figure S1. Coverage of six structural alignment algorithms used in the scaffolding meta-server in the identification of protein scaffolds for the HIV-1 MPER epitope recognized by a broadly neutralizing antibody 2F5 (Ref. 40). The number of scaffolds identified by each individual algorithm is highlighted in blue with the overlap between two algorithms shown in *italic*.

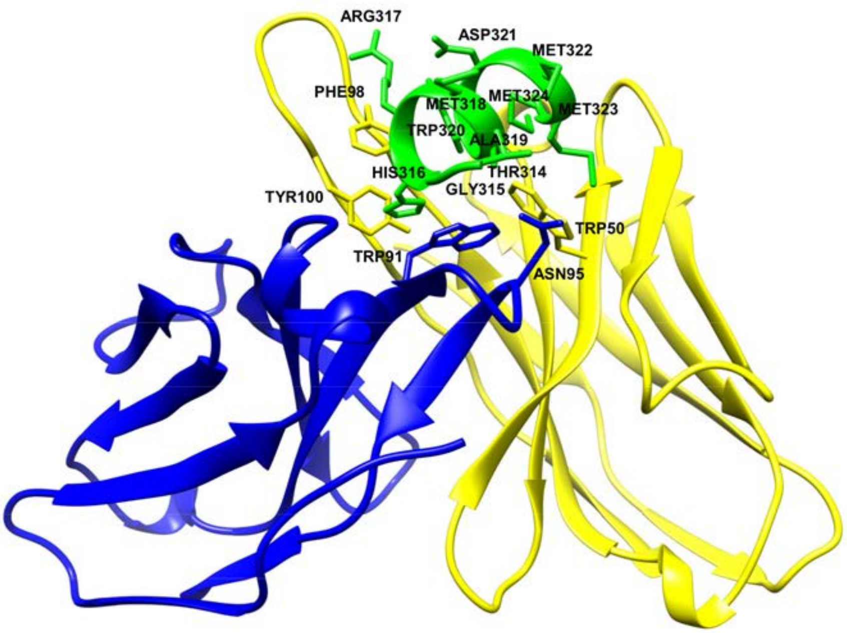

Figure S2. The 11-aa E1 helical epitope (^{314}TGHRMAWDMMM^{324}) bound to the CDRs of murine neutralizing antibody IGH526. Y100E and F98 in HCDR3 interact with H316 and R317 of the epitope, respectively, with further stabilizing contacts provided by W91 and N95 of LCDR3 as well as W50 of FR2.

```
Epitope    TGHRMAWDMMM   Epitope    TGHRMAWDMMM   Epitope    TGHRMAWDMMM
1JKO_C_ES  AGHRIAWLLMM   1LQV_C_ES  CDHAEAWDIMM   1VQO_U_ES  CSHRCAWDAMM
           :***:** :**               .*  ***:**                ** *** **

Epitope    TGHRMAWDMMM   Epitope    TGHRMAWDMMM   Epitope    TGHRMAWDMMM
1XU2_R_ES  APHRCAWDCMM   2F60_K_ES  LSHRAAWDLMM   3CA7_A_ES  CGHRFAWDCMM
             ** *** **                ** ***:**               ***:*** **

Epitope    TGHRMAWDMMM   Epitope    TGHRMAWDMMM   Epitope    TGHRMAWDMMM
3E8Y_X_ES  YGHRCAWDCMM   3F2U_A_ES  DGHRLAWDFMM   3G7l_A_ES  AGHRLAWWMMR
            *** *** **               ***:***:**              :***:** **

Epitope    TGHRMAWDMMM
3R8S_Z_ES  DGHRIAWDIMM
            ***:***:**
```

Figure S3. Sequence alignment of the epitope-matching region after epitope transplantation for E1 epitope (314-324). The alignment was generated using the ClustalW2 web-server (http://www.ebi.ac.uk/Tools/msa/clustalw2/), with asterisk (*****) indicating identical residues, colon (**:**) indicating residues with strongly similar properties, and period (**.**) indicating residues with weakly similar properties.

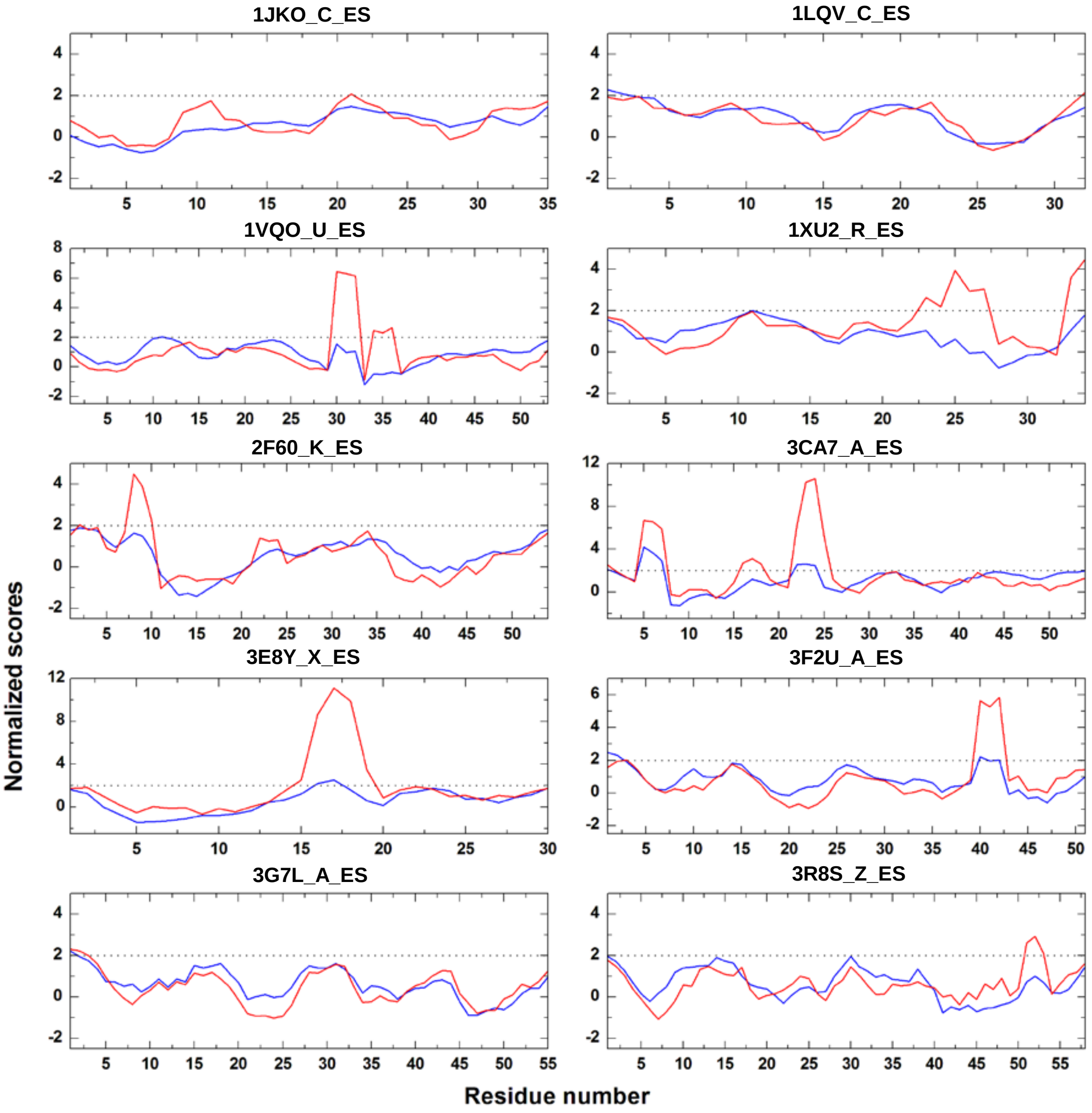

Figure S4. Local quality assessment of the HCV E1 epitope scaffolds. Normalized, residue-based quality scores, calculated from either DFIRE (blue) or a tabulated soft-core vdW function (red) are plotted as a function of residue number (Ref 55). A quality cutoff of 2.0 is shown as black dotted line. Any residues with a quality score above 2.0 would be subjected to visual inspection for further optimization.

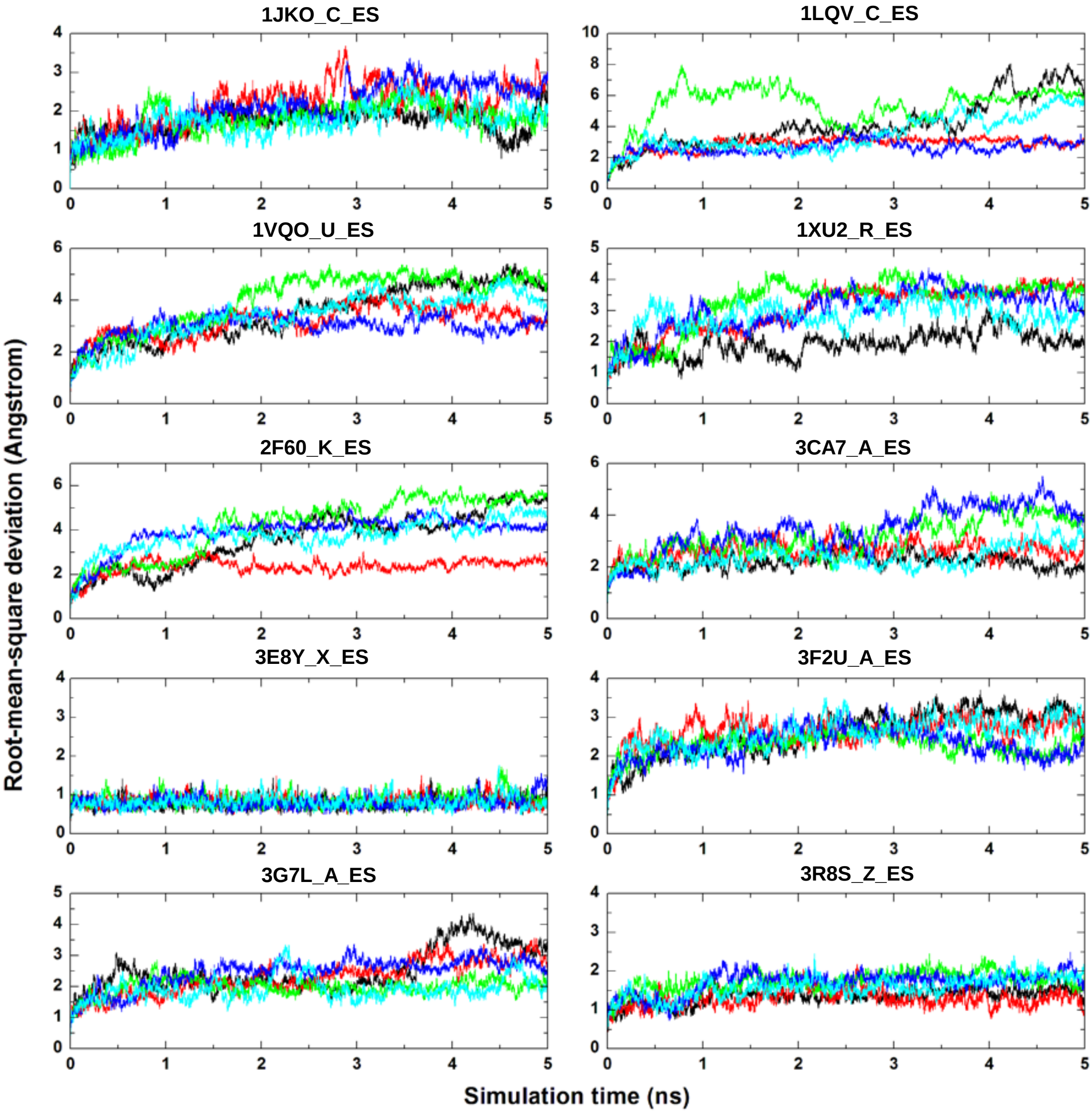

Figure S5. Root-mean-square deviation (RMSD) of Cα atoms during explicit-water molecular dynamics (MD) simulation for 10 HCV E1 epitope scaffolds. RMSD is calculated as the average distance between Cα atoms after superposition of MD-generated conformation onto the initial model. For each epitope scaffold, 5 independent MD simulations, 5 nanoseconds (ns) each, are performed with different initial atomic velocities to sample the conformational space around the initial model.

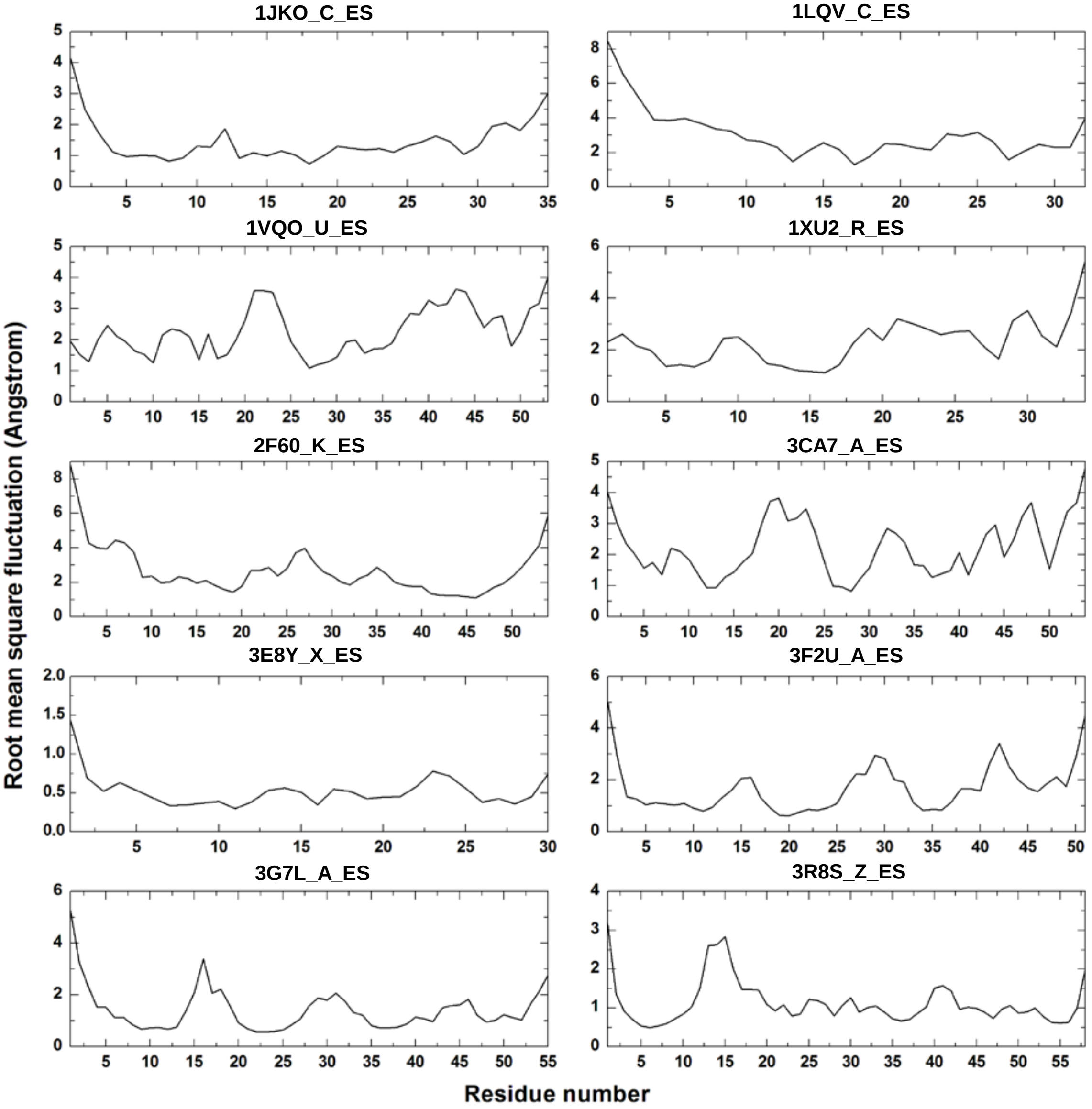

Figure S6. Root-mean-square fluctuation (RMSF) of Cα atoms during explicit-water molecular dynamics (MD) simulation for 10 E1 epitope scaffolds. RMSF is calculated as the fluctuation around the average conformation determined from the conformational ensemble generated by MD simulation. For each epitope scaffold, 5 independent MD simulations, 5 nanoseconds (ns) each, are performed with different initial atomic velocities to sample the conformational space around the initial model. The average conformation is determined from a total of 25,000 conformations.

```
Epitope     QLINTNGSWHIN
2KNM_A_ES   KLINTNGSWHIV
3CA7_A_ES   VLINTNGSWHIV
             ********** 
```

```
Epitope     QLINTNGSWHIN
2YWK_A_ES   GLINLNGSWIIV
3S7R_A_ES   GLHNLNGSWIIP
4F25_A_ES   GLMNLNGSWVIV
             * * ****  * 
```

```
Epitope     QLINTNGSWHIN
2X1F_A_ES   GLYNLNGSWLIC
3P3D_A_ES   GLANFNGSWLLV
             * * **** :  
```

```
Epitope     QLINTNGSWHIN
1T07_A_ES   RLVNCNGSWLIG
            :*:* **** *.
```

```
Epitope     QLINTNGSWHIN
3I8Z_A_ES   KLRNRNGSWEIL
            :* * ****.* 
```

```
Epitope     QLINTNGSWHIN
3L9A_X_ES   SLYNFNGSWYIK
            .* * ****:*:
```

Figure S7. Sequence alignment of the epitope-matching region after epitope transplantation for E2 epitope (412-423). The alignment was generated using the ClustalW2 web-server (http://www.ebi.ac.uk/Tools/msa/clustalw2/), with asterisk (*****) indicating identical residues, colon (**:**) indicating residues with strongly similar properties, and period (**.**) indicating residues with weakly similar properties.

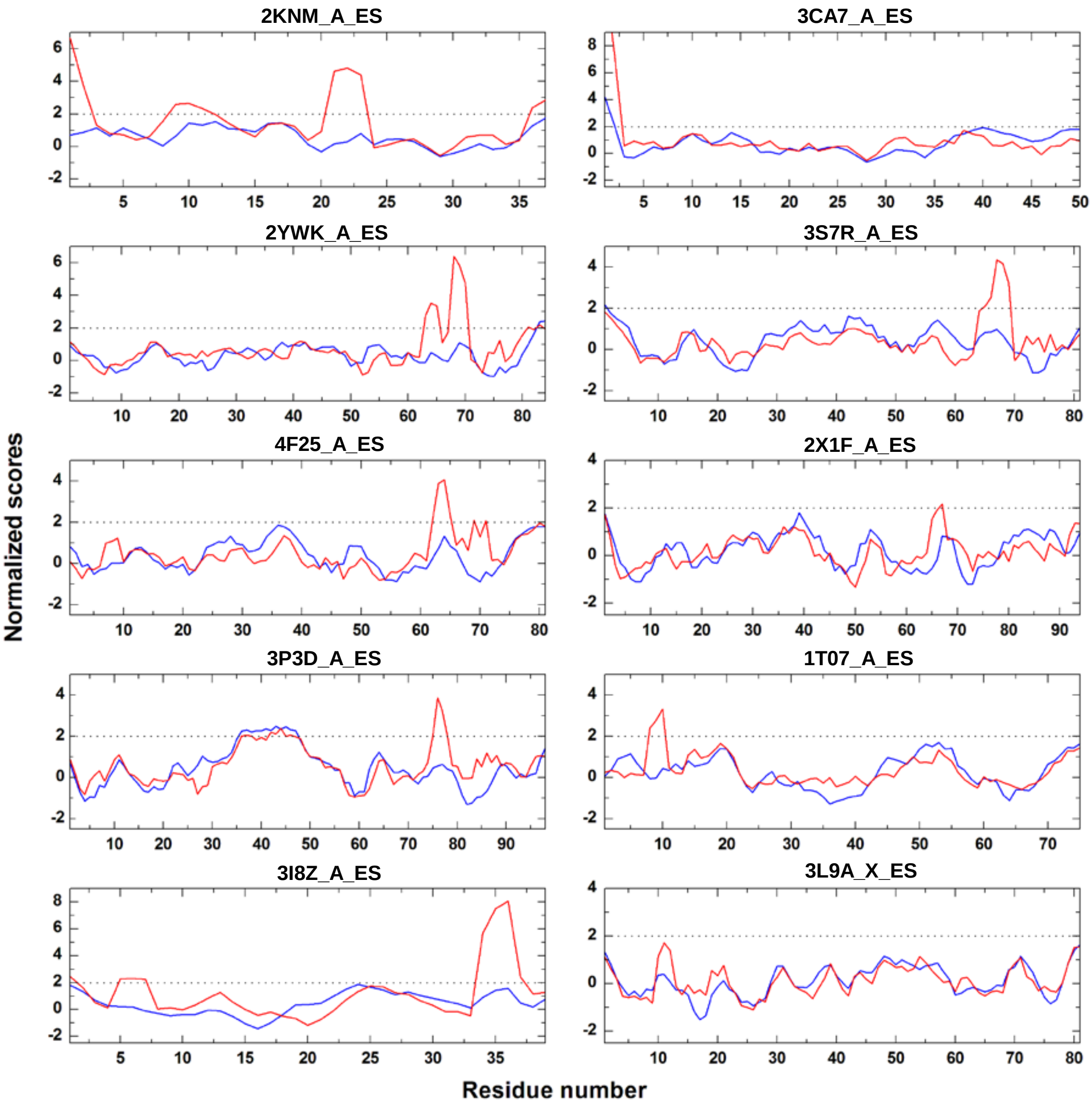

Figure S8. Local quality assessment of the HCV E2 epitope scaffolds. Normalized, residue-based quality scores, calculated from either DFIRE (blue) or a tabulated soft-core vdW function (red) are plotted as a function of residue number (Ref 55). A quality cutoff of 2.0 is shown as black dotted line. Any residues with a quality score above 2.0 would be subjected to visual inspection for further optimization.

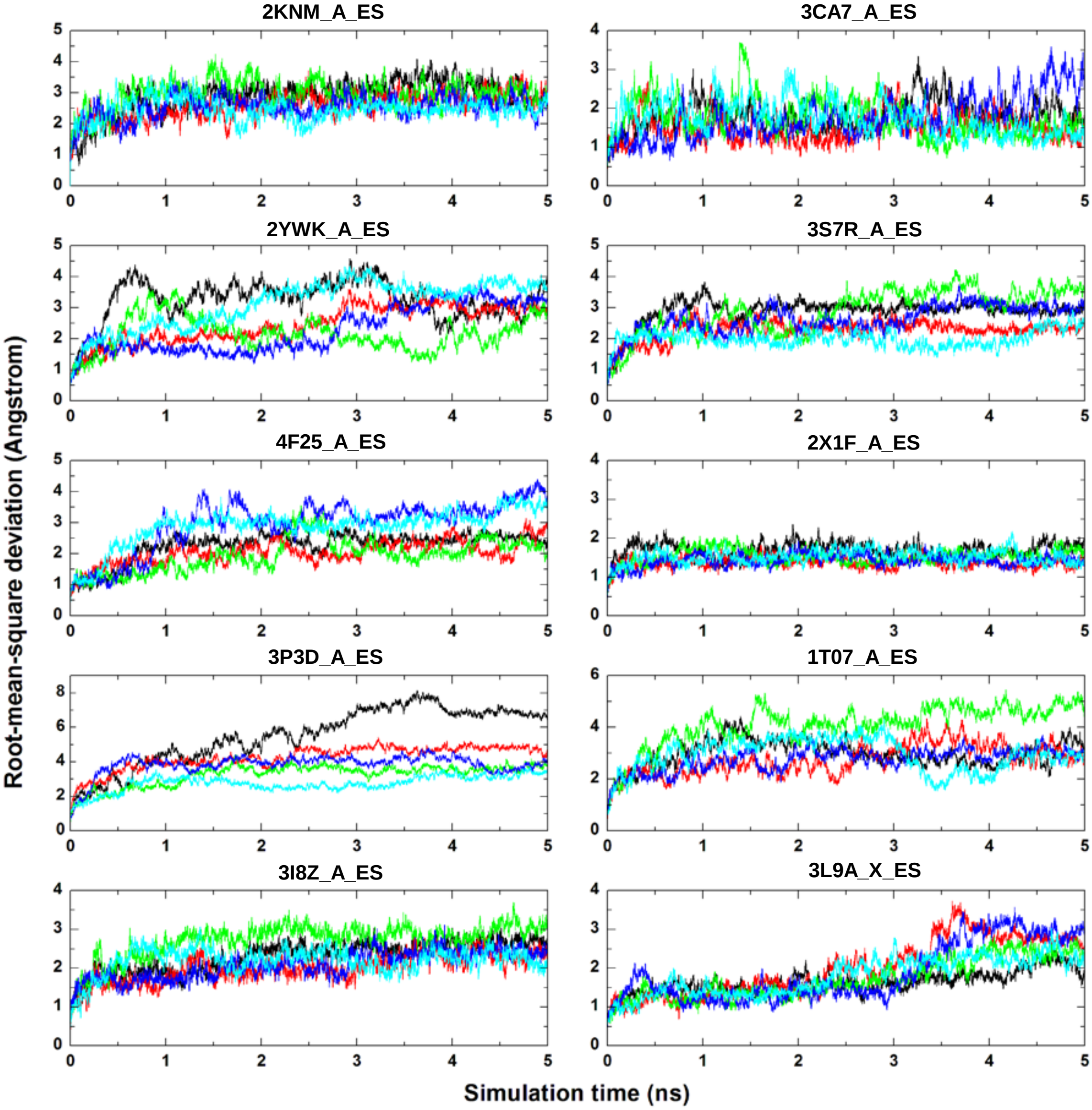

Figure S9. Root-mean-square deviation (RMSD) of Cα atoms during explicit-water molecular dynamics (MD) simulation for 10 HCV E2 epitope scaffolds. RMSD is calculated as the average distance between Cα atoms after superposition of MD-generated conformation onto the initial model. For each epitope scaffold, 5 independent MD simulations, 5 nanoseconds (ns) each, are performed with different initial atomic velocities to sample the conformational space around the initial model.

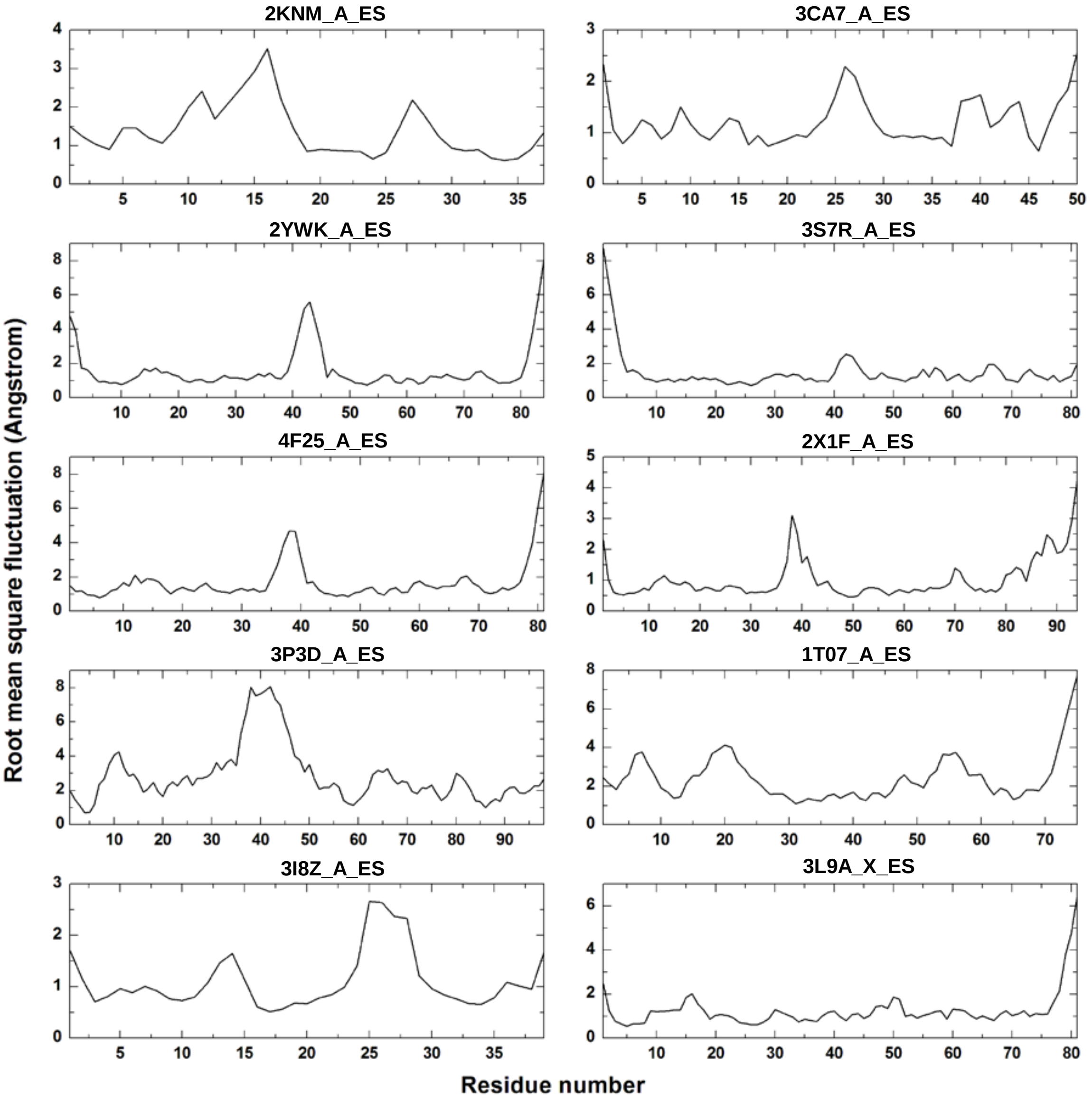

Figure S10. Root-mean-square fluctuation (RMSF) of Cα atoms during explicit-water molecular dynamics (MD) simulation for 10 E2 epitope scaffolds. RMSF is calculated as the fluctuation around the average conformation determined from the conformational ensemble generated by MD simulation. For each epitope scaffold, 5 independent MD simulations, 5 nanoseconds (ns) each, are performed with different initial atomic velocities to sample the conformational space around the initial model. The average conformation was determined from a total of 25,000 conformations.

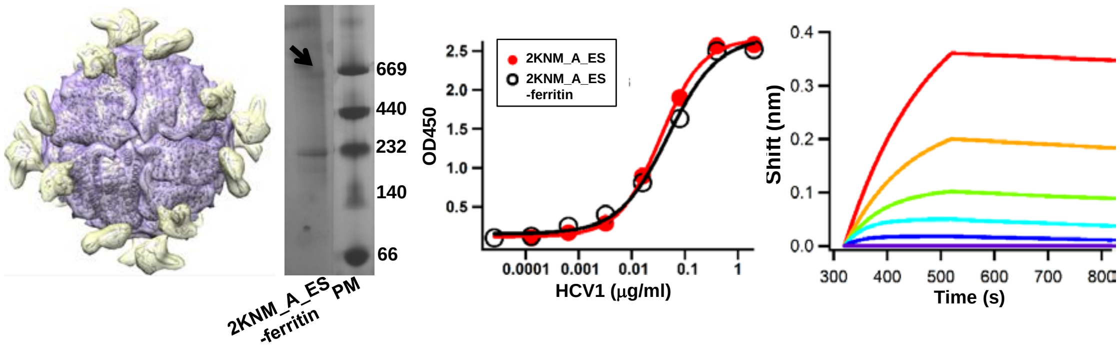

Figure S11. Design and characterization of nanoparticle antigen for the E2 epitope scaffold 2KNM_A_ES. The modeled ferritin nanoparticle displaying twenty-four copies of the antigen (left), blue native (BN) PAGE of the nanoparticle (the second column), ELISA binding of individual epitope scaffold and its particle form to the respective antibody (the third column), and Octet measurement of the nanoparticle (right) are shown. The molecular surface of the nanoparticle model is color-coded differently for ferritin and attached epitope scaffold. Coomassie blue staining was used after BN PAGE. HCV1 was used in ELISA and Octet experiments to test the antibody binding of the E2 nanoparticle. The sensorgrams show the nanoparticle-antibody binding using an antigen titration series of six starting at the maximum concentration of 10μg/ml (calculated based on a single subunit) with a two-fold dilution. The antibody concentration was fixed at 1μg/ml.

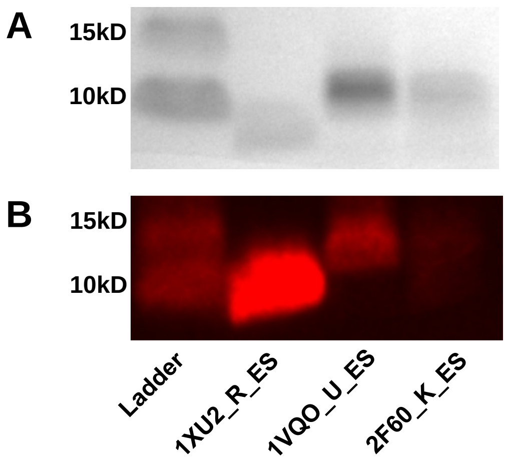

Figure S12. Immunoblot analysis of purified E1 epitope scaffolds (residue 314-324). (A) Reduced SDS-PAGE analysis; (B) Immunoblot analysis: detected by anti-His tag antibody (red). Samples (3µg for SDS-PAGE; 1µg for Immunoblot) were reduced (treatment with 25 mM DTT and heated at 100°C for 5 minutes) and analyzed by SDS-PAGE. For immunoblot, after gel electrophoresis, the resolved E1 scaffolds were transferred to an Immobilon-FL membrane (PVDF type; Millipore) using a semi-dry blotting device (Bio-Rad). The membrane were blocked with the Odyssey Blocking Buffer (LI-COR Biosciences). The immobilized E1 Epitope Scaffolds were detected with 6x-His Epitope Tag Antibody (Thermo Fisher) and IRDye700DX goat anti-mouse IgG secondary antibody (diluted 1:10,000; LI-COR Biosciences). The immunoblot was analyzed with the Odyssey Infrared Imaging System and Image Studio software (LI-COR Biosciences).

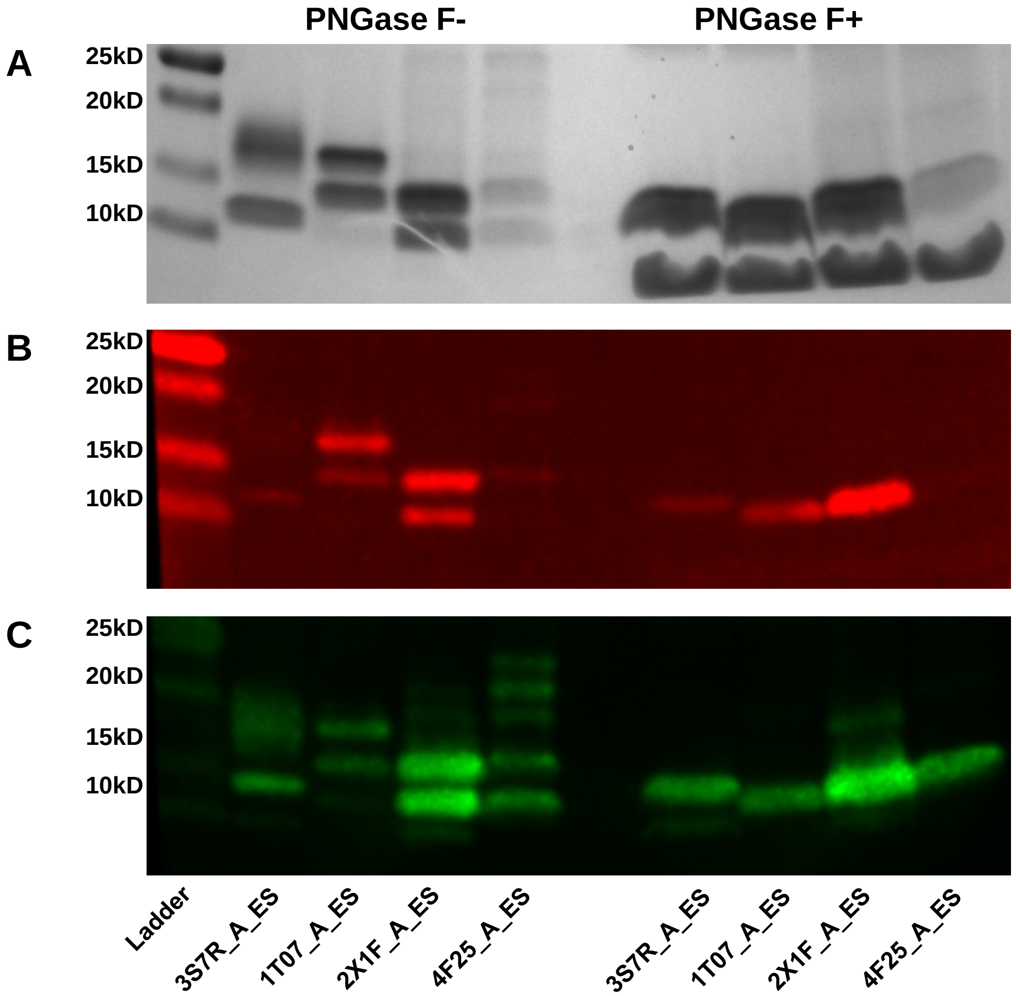

Figure S13. Immunoblot analysis of PNGase F-treated purified E2 epitope scaffolds (residues 412-423). (A) Reduced SDS-PAGE analysis; (B) Immunoblot analysis: mAb HCV1 detection (red); (C) 6x-His Epitope Tag Antibody detection (green). Samples (5µg for SDS-PAGE; 1µg for Immunoblots) were treated with PNGase F (New England Biolabs) prior to being reduced (treatment with 25 mM DTT and heated at at 100°C for 5 minutes) and analyzed by SDS-PAGE. A set with no PNGase F treatment was included as control. For immunoblots, after gel electrophoresis, the resolved E2 scaffolds were transferred to an Immobilon-FL membrane (PVDF type; Millipore) using a semi-dry blotting device (Bio-Rad). The membrane were blocked with the Odyssey Blocking Buffer (LI-COR Biosciences). The immobilized E2 Epitope Scaffolds were detected with mAb HCV1 and IRDye700DX anti-human IgG (diluted 1:10,000; LI-COR Biosciences) (B), or 6x-His Epitope Tag Antibody (Thermo Fisher) and IRDye800CW goat anti-mouse IgG secondary antibodies (diluted 1:10,000; LI-COR Biosciences) (C), respectively. The immunoblots were analyzed with the Odyssey Infrared Imaging System and Image Studio software (LI-COR Biosciences).
